# Stepwise-Enhanced Tumor Targeting of Near-Infrared Emissive Au Nanoclusters with High Quantum Yields and Long-Term Stability

**DOI:** 10.1021/acs.analchem.2c02717

**Published:** 2022-09-15

**Authors:** Hui Zhu, Yue Zhou, Yu Wang, Suying Xu, Tony D. James, Leyu Wang

**Affiliations:** † State Key Laboratory of Chemical Resource Engineering, College of Chemistry, Beijing University of Chemical Technology, Beijing 100029, China; ‡ Department of Chemistry, University of Bath, Bath BA2 7AY, U.K.

## Abstract

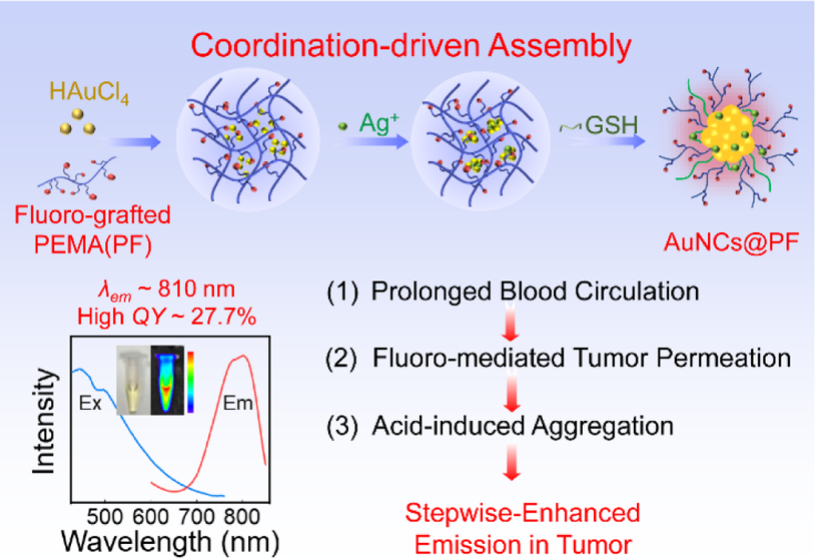

We developed an *in situ* coordination-driven spatially confined strategy for preparing near-infrared emissive gold nanoclusters encapsulated by fluorinated polymers (AuNCs@PF, λ_max_ = 810 nm) with good stability and high quantum yields (27.7%), far higher than those previously reported for NIR AuNCs (>800 nm). Based on the stepwise enhancements including long blood circulation-induced passive tumor targeting, fluoro-enhanced tumor permeation, and tumor microenvironment (weak acid)-induced aggregation retention in cells, these AuNCs demonstrated bright and stable NIR fluorescence imaging ability in tumors. Additionally, the AuNCs@PF were capable of fluorine magnetic resonance imaging and computed tomographic imaging. The multimodal imaging of tumor-bearing mice clearly implied the potential of AuNCs@PF in biomedical fields.

## Introduction

Gold nanoclusters (AuNCs) are a new type of molecule-like luminophores composed of a few to dozens of Au atoms.^1,2^ The differing atomic structure of gold nanoparticles confers unique physicochemical properties on AuNCs, including adjustable photoluminescence and catalytic activity, resulting in great potential for bioimaging, biosensing, and therapeutic applications.^3−7^ In particular, for bioimaging, the luminescent brightness and targeting ability of the probes are essential properties that need to be optimized. The photoluminescence of AuNCs is closely related to the core sizes of the AuNCs, choice of ligands, and coverage densities of ligands.^8,9^ In addition, the introduction of other metal elements^10−12^ and the promotion of charge transfer between the ligands and metals^13,14^ can enhance the photoluminescence efficiency. Alternatively, the creation of a rigid environment by spatial confinement by proteins,^15−17^ polymers,^18−21^ or the assembly of NCs^22−24^ can also promote photoluminescence due to the restriction of non-radiative relaxations. Despite significant progress, AuNCs that were prepared under aqueous system, exhibiting near-infrared (NIR) emission, still suffer from low quantum yields. Thus, it is highly desirable to rationally adjust the emission peak over 800 nm and improve the luminescence brightness and stability of AuNCs.

In order to achieve enough brightness at target sites, sufficient accumulation of nanoprobes is required in addition to optimizing the luminescence intensities of a single luminophore. To this end, increasing the permeation and retention of luminophores at the area of interest is particularly important. In this regard, modification of the surface moieties of nanoprobes with targeting peptides,^25−28^ adoption of charge-switching moieties (switching from negative to positive under acidic conditions) to enhance electrostatic interaction,^29−31^ and increasing cross-membrane ability^32−36^ have been demonstrated. In addition, *in situ* self-aggregation of probes, where the small molecular probes could transfer into larger assemblies or aggregates, has been used to prolong retention in a tumor microenvironment.^37−42^ However, few systems have focused on the retention of AuNCs at tumor sites, partially due to the ultrasmall size of AuNCs. As such, it remains challenging to prepare AuNCs concurrently with high quantum yields and outstanding specificity toward tumor sites.

**Scheme 1:**
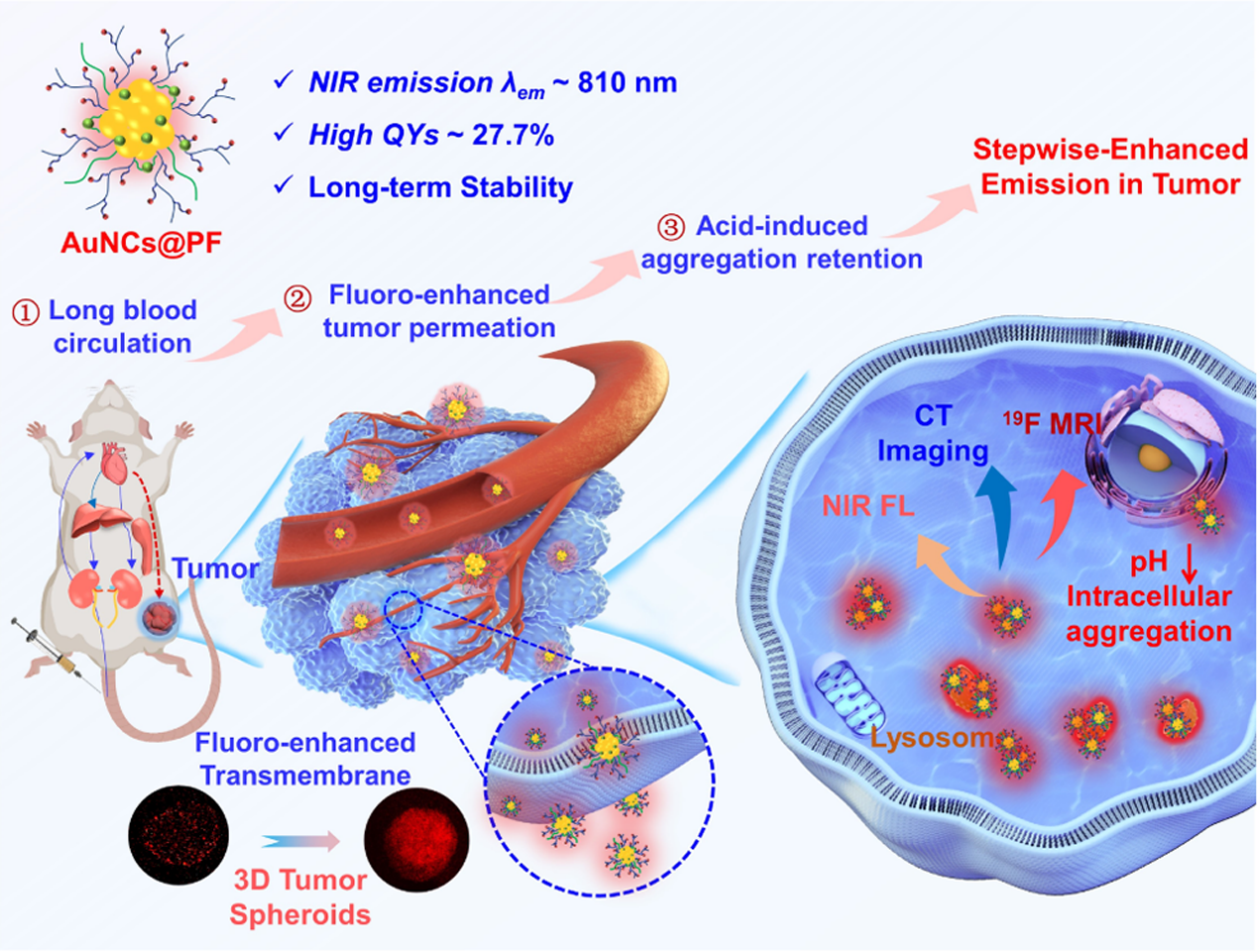
Illustration of AuNCs@PF With Stepwise-Enhanced Emission in Tumors The stepwise-enhanced emission: (1) long blood circulation, (2) fluoro-enhanced tumor permeation, and (3) tumor microenvironment (weak acid)-induced aggregation retention. As shown in 3D tumor spheroids, the AuNCs@PF demonstrated better tumor-permeation ability than that of AuNCs@PE (without fluorine in the functional polymer).

Herein, we fabricated AuNCs embedded in a fluorinated polymer (AuNCs@PF) which exhibited relatively high photoluminescent quantum yields (27.7%) with a maximal wavelength at 810 nm, excellent photostability, and enhanced specificity toward tumor sites *via* spatial confinement in a fluorinated polymer (PF) matrix. The luminescence originated from the high ratio of the Au(I)-thiolate complex, which was stabilized and limited by the numerous carboxyl groups on the polymer. Meanwhile, the good stability and long blood circulation, the improved permeation of fluorinated sidechains toward cell membranes, as well as the weak acid-induced aggregation retention in tumor cells, afforded enhanced emission in tumors (Scheme 1). The NIR fluorescence, X-ray attenuation coefficient of Au, and high loading of fluorine render these AuNCs@PF the *in vivo* imaging ability including NIR fluorescence (FL) imaging, computed tomography (CT) imaging, and fluorine magnetic resonance imaging (^19^F MRI).

## Experimental Section

### Synthesis of 2,2,2-Trifluoroethylamine and Cysteamine-Grafted PEMA (PF)

First, 3 mL of DMF containing PEMA (1 mmol) was added dropwise into 20 mL of water containing 2,2,2-trifluoroethylamine (4 mmol). The mixture solution was stirred at room temperature for 1 h. Next, 10 mg of mPEG-NH_2_ dissolved in 1 mL of water and 225 mg of cystamine dihydrochloride deacidified by 2 mL of NaOH aqueous solution (2 M) were added. After stirring at room temperature overnight, the solution was dialyzed with ultrapure water using a dialysis bag (8000–14,000 Da) for 2 days to remove excess reactants. The purified product was condensed and stored for later use. The functional polymer without fluorine (PE) was prepared by the same method but by replacing the 2,2,2-trifluoroethylamine with ethylamine.

### Quantitation of Fluorine Content in PF

Herein, CF_3_COONa was used as a standard compound for establishing the standard curve of the ^19^F NMR peak area *versus* fluorine concentration. First, CF_3_COONa solution with different concentrations (0.5, 1, 5, 10, 20, and 50 mM) was prepared to test their ^19^F NMR peak area. Then, the linear equation of the ^19^F NMR peak area versus fluorine concentration was calculated to be *A*_peak area_ = 3.9 × 10^6^*C*_F_/mM + 1.3 × 10^7^. The ^19^F concentration of the as-prepared PF (3 mg/mL, ^19^F NMR peak area 1.6 × 10^8^) was calculated to be 35.5 mM (0.674 mg/mL) according to the standard curve. Thus, the mass fraction of ^19^F in polymeric PF was 22.5%.

### Synthesis of AuNCs@PF and AuNCs@PE

All glassware and magnetic stir bars were cleaned using freshly prepared aqua regia and rinsed with ultrapure water three times. In a typical synthesis process, 28 mg of the PF was dissolved in 6 mL of ultrapure water. And then, 100 μL HAuCl_4_ solution (10 mM) was added into polymer solution and stirred at room temperature for 10 min. The pH of the mixture was adjusted with NaOH to 9.0 to form a Au–polymer complex and heated to 80 °C. After stirring for 2 h, 100 μL of AgNO_3_ solution (10 mM) was added into the resulting solution and heated for another 1 h. Next, 40 μmol of GSH dissolved in 2 mL of water was added and heated for another 2 h to make AuNCs@PF. To explore the impact of reaction conditions on the growth of AuNCs@PF, we tuned the amount of PF (0, 7, 14, 28, 42, and 56 mg), the feed ratio of Ag/Au (0, 0.5, 1, 1.5, and 2), the amount of GSH (0, 10, 20, 30, 40, and 60 μmol), and the pH (6.0, 7.0, 8.0, 9.0, 10.0, 11.0, and 12.0) during the formation of the Au–polymer complex. The obtained AuNCs@PF solution was dialyzed with ultrapure water using a dialysis bag (3500 Da) for 2 days to remove excess reactants. Fabrication of AuNCs@PE was performed under identical optimized experimental conditions except for replacing PF with PE.

### Synthesis of GSH-AuNCs

First, aqua regia was used to clean glassware and magnetic stir bars. Typically, 1.8 mL of HAuCl_4_ solution (10 mM) and 200 μL of AgNO_3_ solution (10 mM) were added into 5 mL of ultrapure water, followed by the addition of 2 mL of GSH solution (20 mM). Then, the solution was heated to 80 °C and stirred for 6 h. The obtained GSH-AuNCs were dialyzed with ultrapure water for 24 h to remove excess reactants.

### Calculation of Fluorescence QYs

The quantum yields (QYs) of the AuNCs@PF were determined in PBS by using ICG dye as a reference standard *via* equations^1,20^
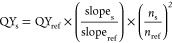
1

where QY_s_ and QY_ref_ are the fluorescence quantum yields of AuNCs@PF in PBS and reference samples (free ICG in PBS), respectively; slope_s_ and slope_ref_ are referred to as the linear fitting slopes of fluorescence integration of AuNCs@PF and ICG *versus* absorption values of AuNCs@PF (450 nm) and ICG (760 nm) under a series of concentrations, respectively; and *n*_s_ and *n*_ref_ are the refractive index values of the solvent. Here, both AuNCs@PF and ICG were prepared in PBS; thus, the ratio of *n*_s_ to *n*_ref_ shall be 1. For the freshly prepared ICG solution in PBS, the QY_ref_ was 2.5%.

### Cell Viability

The cytotoxicity of the as-prepared AuNCs was evaluated using an MTT assay with cultured 4T1 (mouse breast cancer cells). Typically, 4T1 cells (∼5 × 10^4^ cells well^–1^) were seeded in a 96-well microtiter plate, and then different concentrations of AuNCs (from 0–500 μg mL^–1^) were added and cultured at 37 °C for 24 or 48 h under 5% CO_2_ and a 95% relative humidity atmosphere, respectively. After that, 20 μL of sterile-filtered MTT stock solution in PBS (5 mg mL^–1^) was added to each well. The 96-well microtiter plate was incubated at 37 °C for another 4 h. The upper suspension of each well was removed, and then, 120 μL of DMSO was added into each well prior to measuring the absorption of formazan at 492 nm using an ELISA plate reader (F50, Tecan).

### Cell Imaging

4T1 cells were seeded on a sterilized Petri dish and cultured overnight at 37 °C in a 5% CO_2_-humidified incubator. Then, the AuNCs@PF or AuNCs@PE solution was added into the cell culture dish with a final concentration of 200 μg/mL, which was further incubated for another 2, 4, and 8 h, respectively. Thereafter, the cells were washed with phosphate buffer saline and fixed with 4% paraformaldehyde solution for 20 min. Fluorescence imaging was carried out by confocal laser scanning microscopy (CLSM) (Leica SP8) with an excitation wavelength of 488 nm.

### Colocalization Imaging of Lysosomes

4T1 cells were seeded on a sterilized Petri dish and cultured overnight at 37 °C in a 5% CO_2_-humidified incubator. Then, AuNCs@PF solution was added into the cell culture dish with a final concentration of 200 μg/mL and incubated for another 8 h. Thereafter, the cells were washed with PBS and incubated with 100 nM LysoTracker Green DND-26 (ex/em: 504/511 nm) and dissolved in PBS for another 30 min. Finally, the LysoTracker Green was washed away, and cells were fixed with 4% paraformaldehyde by CLSM imaging at the excitation wavelength of 488 nm.

### 3D Multicellular Tumor Spheroid Imaging

4T1 cells with a cell density of 1 × 10^5^ cells were seeded in ultralow adsorption 24-well plates. After being cultured for 3–4 days, tumor spheres with a diameter of 300–400 μm were selected and incubated with AuNCs@PF and AuNCs@PE, respectively, with a concentration of 200 μg/mL. After incubation for 1 h, the tumor spheres were washed twice with PBS and redispersed in 100 μL of DMEM for CLSM imaging.

### Cellular Retention Investigation of AuNCs@PF and GSH-AuNCs

4T1 cells with a cell density of 1 × 10^5^ cells were seeded in 12-well plates. After being cultured for 24 h, AuNCs@PF and GSH-AuNC solutions were added into the well with a final concentration of 200 μg/mL and incubated for another 12, 24, 36, 48, and 72 h, respectively. During incubation, the cells were washed with PBS every 12 h and replenished with fresh cell culture medium. At different time points, the cells were taken off by using trypsin and counted using a cell counter. After that, the cells were lysed with freshly prepared aqua regia for 24 h. The Au content in the samples was then analyzed by inductively coupled plasma mass spectrometry (ICP–MS).

### Animal Model

Animal experiments were performed using Balb/c female mice (4 weeks) and approved by the local ethics review board. All animal experiments were implemented under the guidelines of the Animal Care and Use Committee of the China–Japan Friendship Hospital. 4T1 cells (∼2*10^6^) dispersed in PBS were injected into the right flank of 4 week old female mice subcutaneously. As the tumors grew to around 300 mm^3^, the mice were used for fluorescent imaging.

### Optical Imaging of AuNCs@PF and AuNCs@PE

Balb/c mice were divided into two groups (*n* = 3), and mice in each group were intravenously injected with AuNCs@PF and AuNCs@PE (100 μL, 10 mg/mL), respectively. After 30 min, 1, 2, 4, 12, and 24 h post-injection, the mice were anesthetized with isoflurane, and *in vivo* optical imaging was carried out on the small animal live imaging system from PerkinElmer (IVIS Spectrum) with an excitation wavelength of 465 nm. The optical images were collected under emission at 800 nm.

### Biodistribution of AuNCs@PF

Balb/c mice were divided into six groups (*n* = 3), and each mouse was intravenously injected with solutions of AuNCs@PF (100 μL, 10 mg/mL). After 30 min, 2, 4, 12, 24, and 36 h post-injection, the mice were sacrificed, and whole blood was collected from the orbit; meanwhile, vital organs, including the heart, liver, spleen, lungs, kidneys, and intestines, were excised, rinsed with PBS, and then dried at room temperature for 6 h. Then, the organs and blood samples were immersed in aqua regia and allowed to be digested for 48 h. The Au content in the samples was then analyzed by ICP–MS.

### *In Vitro* and *In Vivo* CT Imaging

The AuNCs@PF solution with different concentrations (0, 1.2, 3, 5, 10, and 20 mg mL^–1^) was prepared for *in vitro* CT imaging. The AuNCs@PF solution (30 mg mL^–1^ in PBS, 100 μL) was intratumorally injected into the tumor-bearing mice. The *in vivo* CT imaging measurements were carried out after the mice were anesthetized with isoflurane. The CT X-ray tomography system (Triumph X-SPECT/X-O CT) was adopted with scanning parameters set as follows: scanning voltage: 65 kV, current: 185 μA; FOV: 90 mm; matrix: 1024 × 1024, scanning layer thickness 1 mm, and layer spacing 1 mm.

### *In Vitro* and *In Vivo* MRI Experiments

The AuNCs@PF solution with different concentrations (12, 24, 30, 36, 48, and 60 mg mL^–1^) was prepared for *in vitro* MRI imaging. The AuNCs@PF solution (30 mg mL^–1^ in PBS, 100 μL) was intratumorally injected into the tumor-bearing mice. The *in vivo* MRI measurements were carried out after live mice were anesthetized with isoflurane. The *T*_1_-RARE sequence was used for ^19^F MRI, and the related parameters were set as follows: the matrix size was 100 × 100; TR and TE were 3000 and 4.64 ms, respectively; and the field of view (FOV) was set at 40 mm × 40 mm with a slice thickness of 5 mm. The total experiment time was 19 min. The *T*_2_-TurboRARE sequence was used for ^1^H MRI, and the related parameters were set as follows: the matrix size was 200 × 200; TR and TE were 4529 and 40 ms, respectively; and the field of view (FOV) was set at 40 mm × 40 mm with a slice thickness of 1 mm. The total experiment time was 3 min 10 s.

## Statistical Analysis

All quantitative data were expressed as mean ± standard deviation (SD). The statistical significance was carried out using one-way analysis of variance (ANOVA) with Origin 2021 (OriginLab).

## Results and Discussion

**Figure 1:**
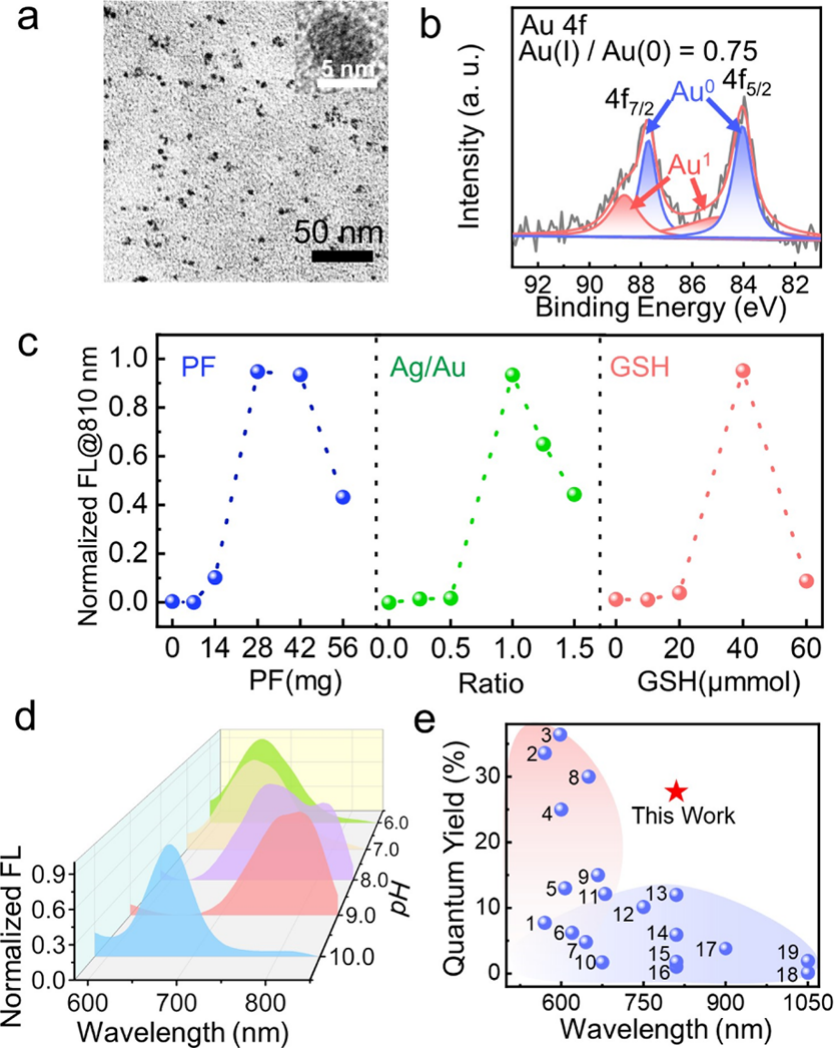
Preparation and characterization. (a) TEM image of AuNCs@PF. Inset is the corresponding HRTEM image. (b) XPS spectra of Au 4f in AuNCs@PF. (c) Normalized emission intensities of AuNCs at 810 nm under different conditions including the amount of PF, feeding ratios of Ag/Au, and GSH dosages. (d) Normalized FL spectra of AuNCs@PF synthesized under different pH conditions. (e) Comparison in quantum yields and maximal emission wavelengths of AuNCs in the literature (the number represents the serial number of references in Table S2) and our work (the red star).

Inspired by our previous work,^21^ rational modulation of the polymer structure was proposed for constructing highly emissive AuNCs with good targeting features. A PF was fabricated by reacting poly(ethylene-*alt*-maleic anhydride) polymer (PEMA) with 2,2,2-trifluoroethylamine and cystamine (Figures S1, S2 and Table S1). As a control, the functional polymer without fluorine (PE) was also synthesized *via* the same protocol but by replacing the 2,2,2-trifluoroethylamine with ethylamine and then used for the fabrication of AuNCs@PE. Figure 1a shows the transmission electron microscopy (TEM) image of AuNCs prepared within the PF (AuNCs@PF), which exhibits good dispersion in water with an average hydrodynamic size of around 7.7 ± 0.8 nm (Figure S3a). The UV–vis spectrum in Figure S3b exhibited no observable localized surface plasmon resonance (LSPR) absorption between 500 and 600 nm, indicating that the gold nanoparticles (AuNPs) were not formed during the *in situ* growth process. X-ray photoelectron spectroscopy (XPS) results for Au 4f (Figure 1b) indicated a high Au(I)/Au(0) ratio of 0.75 in the as-prepared AuNCs@PF, which is particularly beneficial for high luminescence.^7^ Silver species were introduced during the synthesis to enhance the luminescence, yet both intensity enhancement and emission peak shifts were observed in the course of varying Ag/Au ratios, suggesting that partial occupancy of Ag in emissive Au moieties shall also occur. Judging from the XPS results of Ag in Figure S4a, the dominant Ag species was Ag(I) in the optimized AuNCs@PF, suggesting that the Ag(I) mainly acts as a linker for the formation of large Au(I)-thiolate complexes, which is consistent with the element mapping images in Figure S5. Although, due to the high molecular weight of polymers, it is hard to get the exact composition of the nanoclusters. As indicated in Figure 1c, several factors including the amount of PF, Au/Ag ratio, and glutathione (GSH) significantly influenced the luminescence intensity of AuNCs@PF at 810 nm. The PF provides a spatial confinement matrix for the nanoclusters to grow. As shown in Figure S6, simple physical mixtures of either PFs with HAuCl_4_ or PF with HAuCl_4_/AgNO_3_ in the absence of GSH resulted in clear spherical nano-assemblies (without fluorescence), indicating that the polymer matrix as a soft template provides multiple anchoring sites as well as spatial confinement during synthesis. When insufficient PF (≤14 mg) was used, the typical LSPR absorption of AuNPs was observed (Figure S7a), whereas an excess of PF with more bind sites (>56 mg) resulted in the generation of larger nanoclusters, displaying a blue-shift of the emission peak (Figure S7b). An optimal amount of PF was determined to be 28 mg. Ag ions can promote the red-shift of the maximal emission peak as well as enhance the intensity of the AuNCs@PF (Figure S7c) at different Ag/Au ratios, implying that Ag ions participated in nanocluster formation in addition to the cross-linking of Au-thiolate complexes into larger motifs.^14,43,44^ Moreover, unlike the previous research where the coverage densities of GSH on the NC surface tended to inversely change the position of the emission peak, namely, less GSH resulting in a longer emission wavelength,^8,9^ in this case, either more or less GSH would result in a blue-shift of the emission peak of AuNCs@PF (Figure S7d).

In addition, the anchoring abilities of PF and GSH were strongly dependent on the pH, which, in turn, affected the luminescence intensities and emission peaks. As indicated in Figures 1d and S8, with an increase in pH from 6.0 to 9.0, the maximal emission peak gradually shifted to a longer wavelength at a given reaction time; however, when the pH values were over 11.0, the typical LSPR absorption peak of AuNPs appeared (Figure S8i), suggesting that the increase in pH promoted the formation of an Au(0) core. TEM images of AuNCs@PF prepared under different pH values verified that larger AuNCs@PF were obtained under more basic conditions, which would then form nanoparticles at higher pH values (Figure S9). Under optimized conditions (Ag/Au = 1:1; PF = 28 mg; GSH = 40 μmol, pH = 9.0), AuNCs@PFs emitting at 810 nm with a quantum yield (QY) of 27.7% were obtained (Figure S10), where the luminescence performance was superior to most of the reported AuNCs in terms of the emission peak position and intensity (Figures 1e, Table S2).

Moreover, the AuNCs@PF exhibited excellent stability, where the DLS size was well maintained in phosphate buffer saline (PBS) and fetal bovine serum (FBS) solution for over 4 weeks (Figure S11a,b), respectively. More interestingly, the AuNCs@PF possessed good tolerance to biothiols. l-cysteine (l-cys) has been frequently used to coordinate with Ag(I), which would normally induce a significant decrease in the luminescence of metal nanoclusters as well as induce a blue-shift in the emission peak. However, in this research, after incubation with high concentrations of l-cys (1 mM) for 12 h, the peak position exhibited no observable change other than a slight decrease in emission intensity (Figure S11c), indicating that the coordination restricted polymer matrix could effectively protect the luminescent nanoclusters.

Fluorinated polymers have been found to promote delivery of genes and proteins into the cytoplasm due to their low surface energy and lipophobic properties.^33,34,45,46^ We anticipated that a significant amount of fluorine atoms may increase the permeability of AuNCs@PF. In this regard, the cell uptake efficiency of AuNCs@PF was investigated, and as a control, polymer grafted with ethylamine (without fluorinated side chain) was utilized to afford AuNCs@PE (Figure S12) with a similar particle size and shape. Moreover, both AuNCs@PF and AuNCs@PE exhibited roughly the same emission intensity at 810 nm at the same concentration (Figure S13a, 3.5 mg mL^–1^), suggesting that the fluorine moieties impose little effect on the formation of nanoclusters.

**Figure 2:**
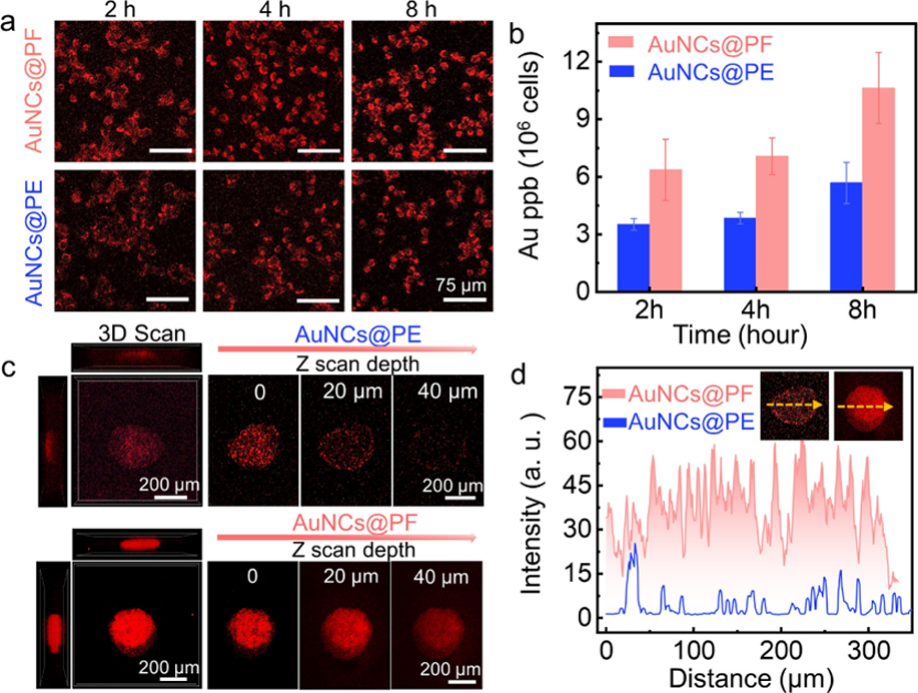
Enhanced penetrability of AuNCs@PF. (a) Confocal fluorescence imaging of 4T1 cells and (b) Au content in cell lysates after incubation with AuNCs@PF and AuNCs@PE for different time intervals, respectively. (c) 3D scan images of tumor spheroids incubated with AuNCs@PE and AuNCs@PF, respectively, obtained with CLSM by a *Z*-stack tomoscan and (d) luminescence intensities for line scanning of 3D tumor spheroids at a given depth. Insets were fluorescence images of tumor spheroids incubated with AuNCs@PE (left) and AuNCs@PF (right), respectively.

As depicted in Figures 2a and S13b, the emission intensities of cells gradually increased with prolonged incubation time, and notably, for cells treated with AuNCs@PF, a significant enhancement in the fluorescence intensity was observed over AuNCs@PE, implying enhanced cellular uptake of AuNCs@PF. These results were further verified by the Au content (ICP–MS results) for cell lysates at different incubation time intervals (Figure 2b), where more Au content existed in cells treated with AuNCs@PF, consistent with the aforementioned observation in fluorescence imaging. Encouraged by the results at the cellular level, 4T1 tumor spheroids were further employed to evaluate the intratumoral penetration behavior of AuNCs@PF and AuNCs@PE, respectively. The 3D scanning images (Figures 2c and S14) revealed that AuNCs@PF could penetrate throughout the 3D tumor spheroids, whereas, for groups treated with AuNCs@PE, fluorescence was merely observed on the edges of the tumor spheroids, indicating improved penetrating ability of AuNCs@PF. Continuous line scanning results showed a significant fluorescence intensity difference between these two groups (Figure 2d), again demonstrating the enhanced permeability of AuNCs@PF. Such enhanced permeability could be derived from the high tendency of AuNCs@PF for cell uptake as well as a small particle size for transcellular transport.^47^

**Figure 3:**
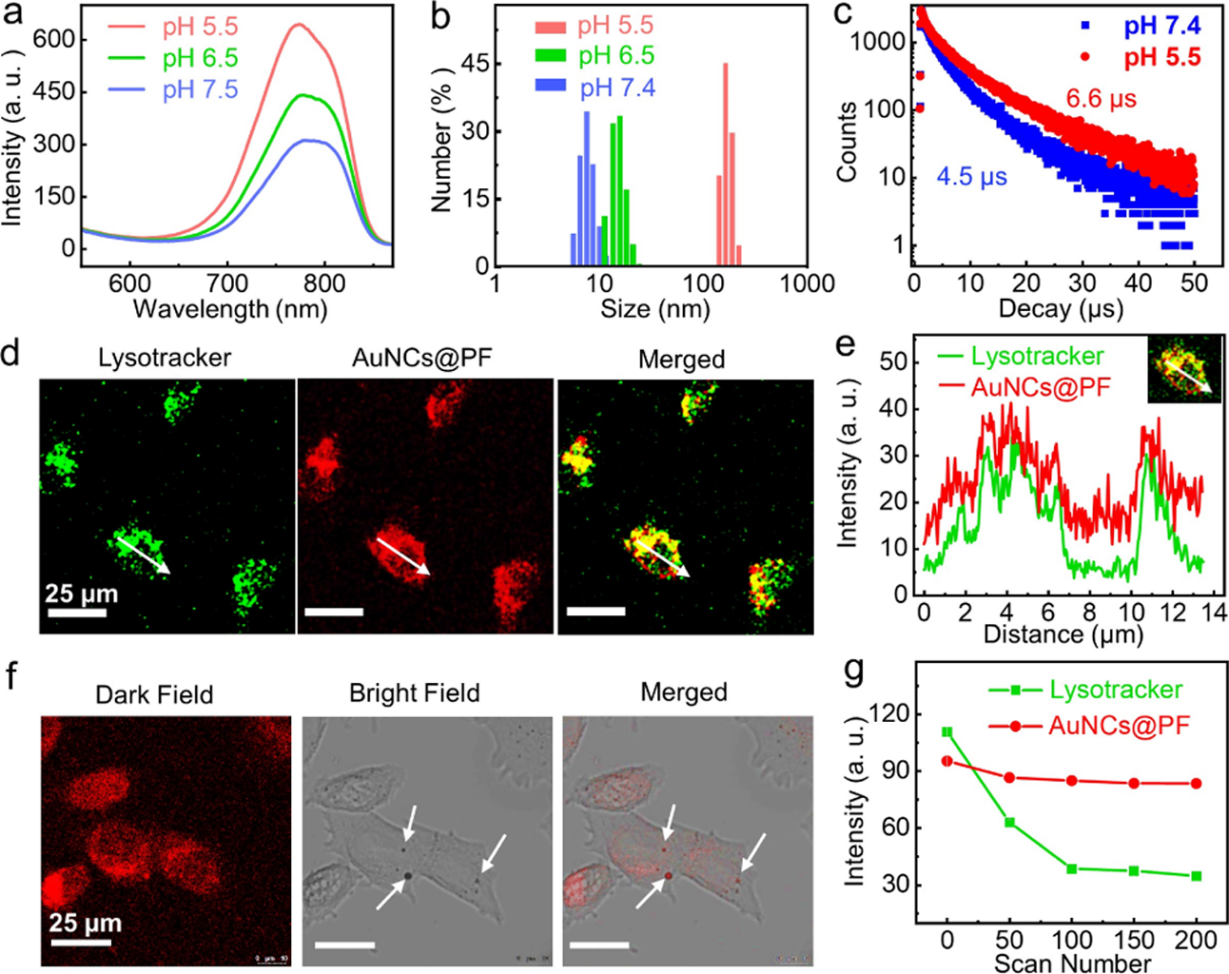
Acid-induced aggregation retention and enhanced emission of AuNCs@PF. (a) Photoluminescence spectra of AuNCs@PF (5 mg mL^–1^). (b) DLS size distribution and (c) fluorescence decay lifetime of AuNCs@PF under different pH conditions. (d) Confocal imaging of 4T1 cells incubated both with AuNCs@PF and LysoTracker (Green DND-26) and (e) the corresponding emission intensities at a given line scanning. (f) Cell imaging of 4T1 cells incubated with AuNCs@PF for 8 h, scale bar = 25 μm. Acid-induced aggregation of AuNCs@PF was highlighted by the arrows. (g) Variations of emission intensities in cells incubated with both LysoTracker and AuNCs@PF under continuous scanning for different times on a confocal microscope.

It was known that AuNC aggregation would induce emission enhancement due to the restriction of non-radiative relaxation of ligand vibration, solvent relaxation, and internal conversion.^13,23,48^ The large amount of carboxyl groups presented on the surface of AuNCs@PF led us to explore the aggregation behavior of AuNCs@PF at different pHs. Clearly, a significant increase in the luminescence intensity was observed under weak acid conditions (Figure 3a). Indeed, both TEM and DLS results suggested the formation of large nanoaggregates of AuNCs@PF with sizes of around 100 nm under pH 5.5 due to the protonation of carboxyl groups on the surface of the AuNCs@PF (Figures S15 and 3b). Meanwhile, the dramatic increase in the luminescence lifetime of AuNCs@PF at pH 5.5 (Figure 3c) further confirmed the acid-induced increase in phosphorescence, which originated from restriction of the Au(I)-thiolate complex. Interestingly, as shown in cell imaging (Figure 3d), for cells treated with AuNCs@PF, there was a high degree of spatial overlap between the red emission of AuNCs@PF and the green fluorescence from commercial LysoTracker Green. It indicated that once internalized into tumor cells, the AuNCs@PF can specifically accumulate in lysosomes, which are known for their acidic nature, with a pH of *ca.* 5.0. These observations were further verified by the line scanning results in one cell (Figure 3e). In addition, clear luminescent dots were observed in Figure 3f, further confirming the aggregation of AuNCs@PF in cells. Such aggregation would significantly increase the size of the system and would prevent the aggregated AuNCs@PF from re-entering circulation and extend the retention in tumor cells, as evidenced by the higher endocytosis efficiency of AuNCs@PF as compared with GSH-AuNCs (Figure S16) and a longer retention time in 4T1 cells with bright fluorescence even after 48 h (Figure S17). Taken together with the improved permeation ability, AuNCs@PF afforded enhanced accumulation at tumor sites in a stepwise manner. Moreover, AuNCs@PF exhibited satisfactory photostability as compared with organic dyes, and no obvious decay in the fluorescence intensity of AuNCs@PF was observed even under continuous 365 nm light excitation for 8 h (Figure S18). Moreover, as shown in the cell confocal fluorescence imaging (Figures S19 and 3g), under continuous scanning for 200 times with 488 nm laser irradiation, a negligible decrease in fluorescence was observed for AuNCs@PF (red emission in Figure S19), whereas the green emission of the commercial lysosome probe (LysoTracker Green DND-26) faded rapidly (Figures S19 and 3g).

**Figure 4:**
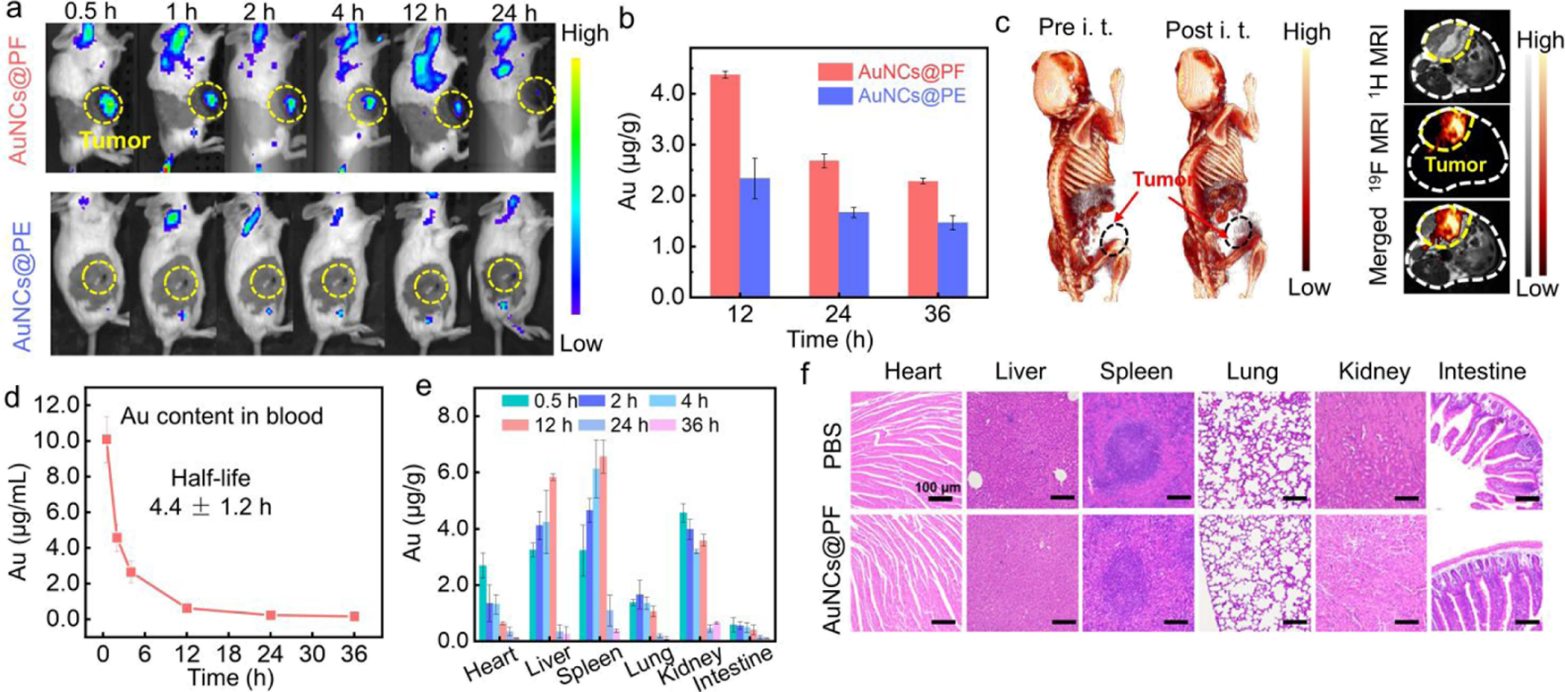
*In vivo* imaging and metabolic studies of AuNCs@PF. (a) *In vivo* imaging of 4T1 tumor-bearing mice after intravenous injection with AuNCs@PF and AuNCs@PE (100 μL, 10 mg/mL), respectively. (b) Time-dependent Au content in tumors of mice treated with AuNCs@PF (*n* = 3) and AuNCs@PE (*n* = 3), respectively. (c) *In vivo* CT imaging of mice before and after the intratumoral injection of AuNCs@PF and ^1^H/^19^F MRI of mice treated with AuNCs@PF. (d) Time-dependent evolution of Au content in blood (*n* = 3). (e) Biodistribution of AuNCs@PF in different organs at different time points (*n* = 3). (f) H&E staining of main organs for mice treated by PBS and AuNCs@PF, respectively. All the Au contents were detected *via* ICP–MS.

Encouraged by the *in vitro*-enhanced penetration and retention ability of AuNCs@PF, we further investigated the *in vivo* imaging performance with Balb/c female mice. Both AuNCs@PF and AuNCs@PE exhibited good cell viability even at concentrations of 500 μg mL^–1^ (Figure S20). After intravenous administration, as shown in Figure 4a, the NIR luminescence at tumor sites was observed within 30 min for the AuNCs@PF group, which could retain about 24 h post-injection (Figure S21). Comparatively, the AuNCs@PE group only exhibited weak emission in the tumor. The quantitative analysis of Au content at tumor sites revealed that AuNCs@PF was more effectively enriched in tumor tissue than AuNCs@PE (Figure 4b, ICP–MS results). On account of a high X-ray attenuation coefficient of the Au element,^49−51^ AuNCs@PF could be used for CT imaging (Figures S22 and 4c). In addition, the numerous fluorine atoms endowed the AuNCs@PF with the potential for ^19^F magnetic resonance imaging (MRI). As presented in Figure S23, the ^19^F MRI intensities are proportional to the concentration of AuNCs@PF, and *in vivo*^19^F MRI was successfully obtained (Figure 4c), indicating the great promise for multimodal imaging.

The dynamic biodistribution and clearance behavior of AuNCs@PF were investigated by monitoring the Au content at different time intervals post intravenous injection (*i.v.*) in mice. Clearly, AuNCs@PF possessed a relatively long circulation half-life (4.4 ± 1.2 h, Figure 4d), significantly longer than that of conventional AuNCs capped by small-molecule ligands.^8,25^ The long blood circulation time increased the possibility of accumulation at tumor sites, consistent with the relatively high Au content in tumor tissue. The Au content in the liver, spleen, and kidneys was higher than that in other organs, implying elimination of AuNCs@PF through both renal clearance and hepatic metabolic pathways (Figure 4e).^52,53^ Moreover, histological hematoxylin and eosin (H&E) staining confirmed that no damage was caused to the major organs (the heart, liver, spleen, lungs, kidneys, and intestines) in groups treated by AuNCs@PF (Figure 4f). The blood biochemical analysis of mice 24 h post i. v. injection (Figures S24 and S25) further confirmed that no apparent toxicity was induced by the AuNCs@PF, indicating good biosafety for biomedical application.

## Conclusions

In summary, the AuNCs@PF was fabricated using a coordination-driven spatially confined synthetic strategy with a PF, which exhibited bright NIR emission (810 nm), high quantum yield (27.7%), and long-term stability, which avoided fast renal clearance and ensured relatively long blood circulation and satisfied metabolic processes. Meanwhile, due to the presence of high levels of fluorine and carboxyl moieties, AuNCs@PF exhibited good tumor tissue permeability and acid-induced aggregation properties, affording stepwise-enhanced tumor targeting and fluorescence imaging. The high X-ray attenuation coefficient and fluorine atoms endowed AuNCs@PF with the ability for *in vivo*^19^F magnetic resonance imaging and CT imaging, indicating the decent potential for multimodal imaging with a high penetration depth.

## Supporting Information Available

^1^H/^19^F NMR spectra of PF; DLS size distribution and absorption spectrum of AuNCs@PF; XPS spectra, element mapping of AuNCs@PF; TEM images and photoluminescence spectra of PF-Au assemblies and Ag-PF-Au assemblies; photoluminescence spectra and absorption spectra of AuNCs@PF prepared under different conditions; TEM images of AuNCs@PF prepared under different pH conditions; excitation, emission spectra of AuNCs@PF and the linear relationship of the integrated fluorescence intensity of AuNCs@PF and ICG; stability of AuNCs@PF; structural, size and optical characterization of AuNCs@PE; photoluminescent spectra of AuNCs@PE and AuNCs@PF and the average luminescent intensity of 4T1 cells treated with AuNCs@PF and AuNCs@PE; CLSM images of 3D tumor spheroids under different treatment; TEM images of AuNCs@PF under various pH conditions; cellular uptake of AuNCs with different ligands; cell imaging of AuNCs@PF prolonged incubation time; photostability of lysosome probe LysoTracker Green DND-26 and AuNCs@PF; intracellular photostability of lysosome probe and AuNCs@PF; cell viability of AuNCs@PF and AuNCs@PE; fluorescent images of different organs and tumor; *in vitro* CT images of AuNCs@PF and the linear relationship; *in vitro*^19^F/^1^H MRI images of AuNCs@PF and the linear relationship; blood biochemical and hematological analysis of the mice; and liver and kidney function evaluation (PDF)
