# Supplementary material for: Stepwise-Enhanced Tumor Targeting of Near-Infrared Emissive Au Nanoclusters with High Quantum Yields and Long-Term Stability
Source: Anal Chem. 2022 Sep 15;94(38):13189–96. doi: 10.1021/acs.analchem.2c02717 (PMC9591319; doi:10.1021/acs.analchem.2c02717)
Supplement: Supplementary file 1 — ac2c02717_si_001.pdf [file ac2c02717_si_001.pdf]

Supporting information

Stepwise-Enhanced Tumor Targeting of Near-infrared Emissive Au Nanoclusters with High Quantum Yields and Long-Term Stability

Hui Zhu, [1] Yue Zhou, [1] Yu Wang, [1] Suying Xu,[1]* Tony D. James[2]* and Leyu Wang[1]*

[1] State Key Laboratory of Chemical Resource Engineering, College of Chemistry, Beijing University of Chemical Technology, Beijing 100029, China.

[2] Department of Chemistry, University of Bath, BA2 7AY, Bath, United Kingdom.

*syxu@mail.buct.edu.cn (Suying Xu.); t.d.james@bath.ac.uk (Tony D. James); lywang@mail.buct.edu.cn (Leyu Wang)

Table of Contents

Supporting Figures S2

Supporting Tables S12

References S15

Supporting Figures

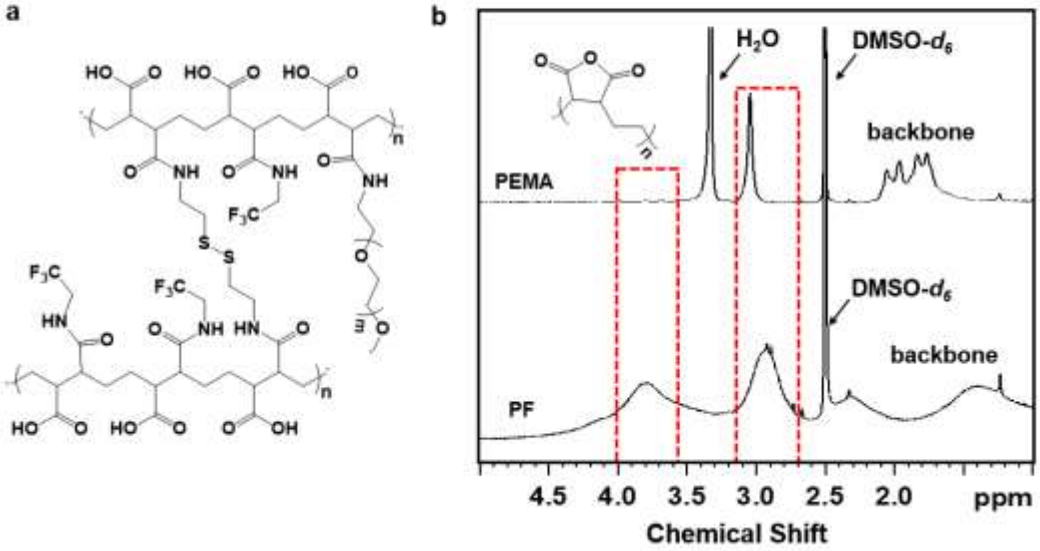

Figure S1. (a) The structure of fluorinated PEMA (PF). (b) ^{1}H NMR spectra of PEMA (top) and fluorinated PEMA (PF, bottom) in DMSO-*d*6. poly(ethylene-alt-maleic anhydride) polymer (PEMA).

The chemical structure of polymer PF was presented as in **Figure S1a**. Compared with the ^{1}H NMR spectrum of PEMA (**Figure S1b**), the new peak was observed at 3.8 ppm, which can be attributed to the proton peak from $–CH_2–$ in trifluoroethylamine. Meanwhile, the peak located at 3.1 ppm, which is the proton of maleic anhydride in PEMA, moved to 2.9 ppm, indicating the occurrence of ring-opening reaction.

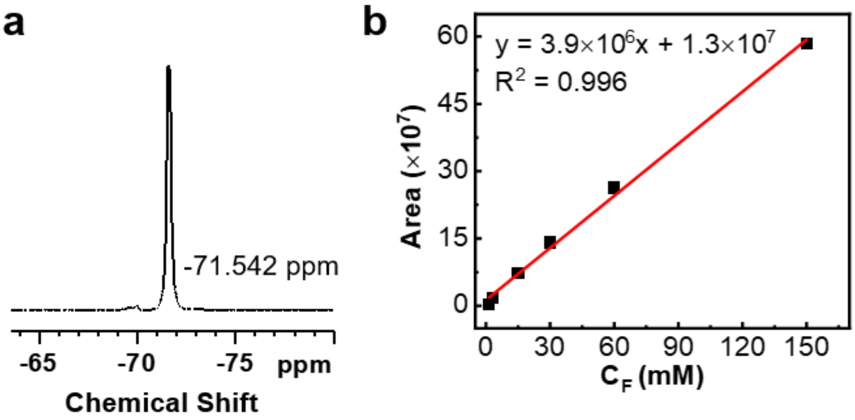

Figure S2. (a) ^{19}F NMR spectrum of polymer PF in H_2O. (b) Plot of the ^{19}F NMR integral peak area *versus* ^{19}F concentration (C_F/mM) by using CF_3COONa as standard solution.

The ^{19}F concentration of the as-prepared PF (3 mg/mL) could be calculated to be 35.5 mM (0.674 mg/mL) by utilizing CF_3COONa as the external standard. Thus, the mass fraction of ^{19}F in polymer PF was 22.5%.

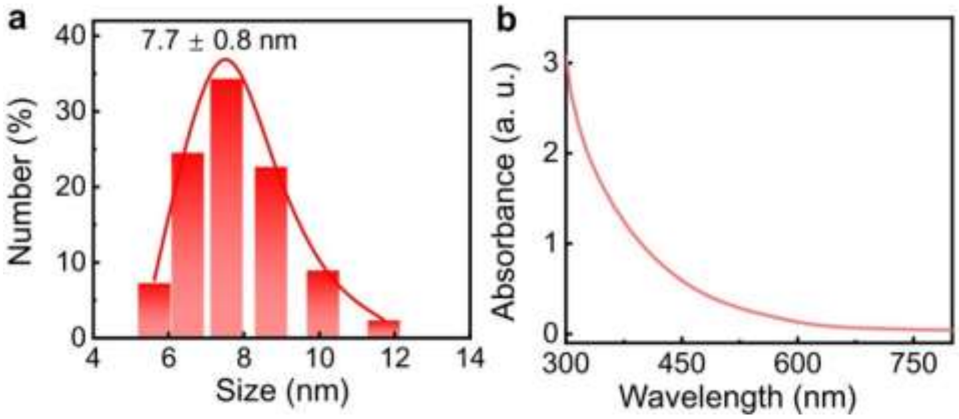

Figure S3. (a) Dynamic lighter scattering (DLS) size distribution and (b) absorption spectrum of AuNCs@PF.

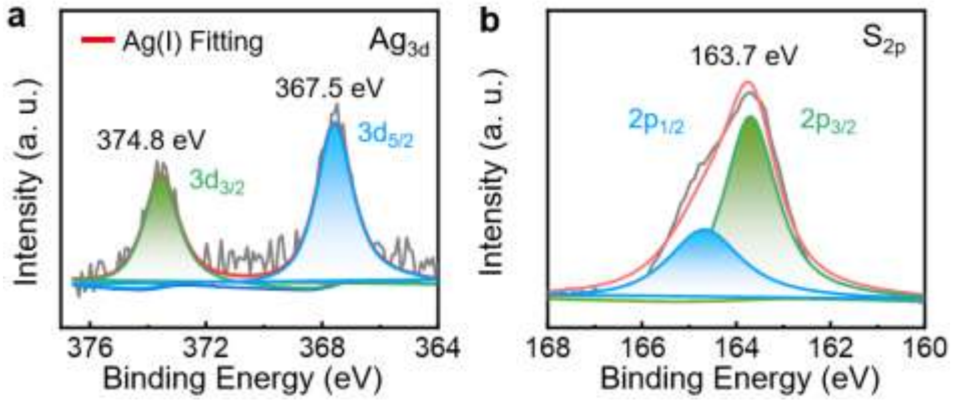

Figure S4. The XPS spectra of (a) Ag 3d and (b) S 2p in the as-prepared AuNCs@PF.

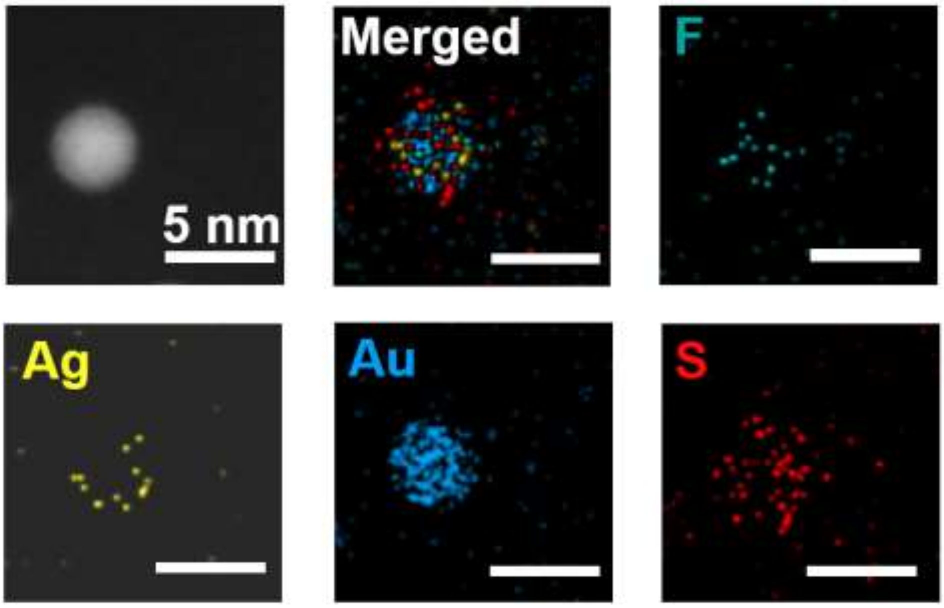

Figure S5. HAADF-STEM images of AuNCs@PF and corresponding elemental mapping. The elemental mapping results suggested that Ag element was mainly located around the surface of AuNCs@PF, which may act as a linker for the formation of AuNCs@PF.

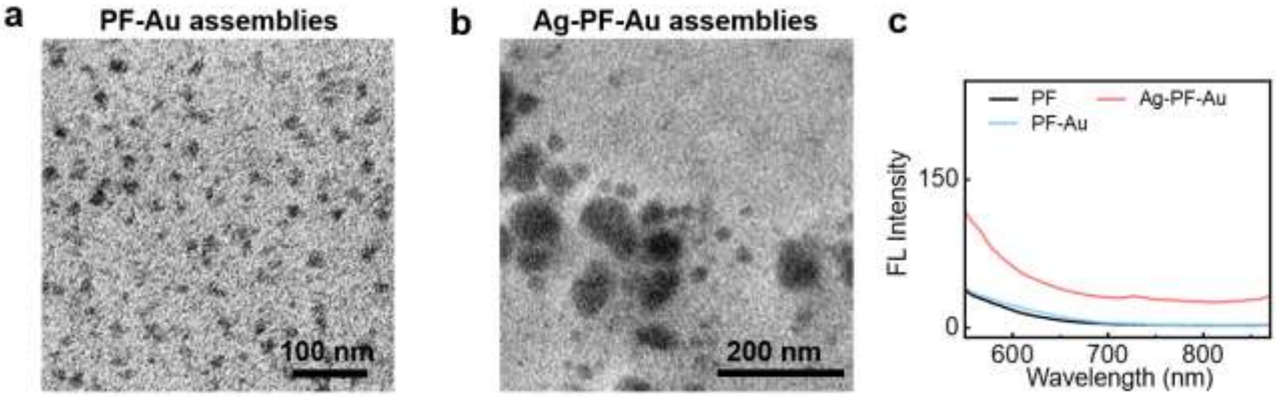

Figure S6. TEM images of (a) PF-Au assemblies and (b) Ag-PF-Au assemblies. (c) Photoluminescence spectra of PF, PF-Au assemblies and Ag-PF-Au assemblies. The peak located at 532 nm can be attributed to the autofluorescence of PF. Note, the FL spectra of PF and PF-Au totally coincide with each other, suggesting no luminescent nanoclusters were formed under these conditions. The fabrication of PF-Au assemblies and Ag-PF-Au assemblies are as follows: 100 µL of $HAuCl_4$ solution (10 mM) was added into 5 mL solution containing 28 mg of PF at room temperature. The pH value of the mixture was adjusted with NaOH to 9.0 to form Au-polymer complex, and then the mixture was heated to 80 °C and stirred for 2 h to form PF-Au assemblies .100 µL of $AgNO_3$ solution (10 mM) was further added into the PF-Au assemblies solution and heated for another 1 h to form Ag-PF-Au assemblies.

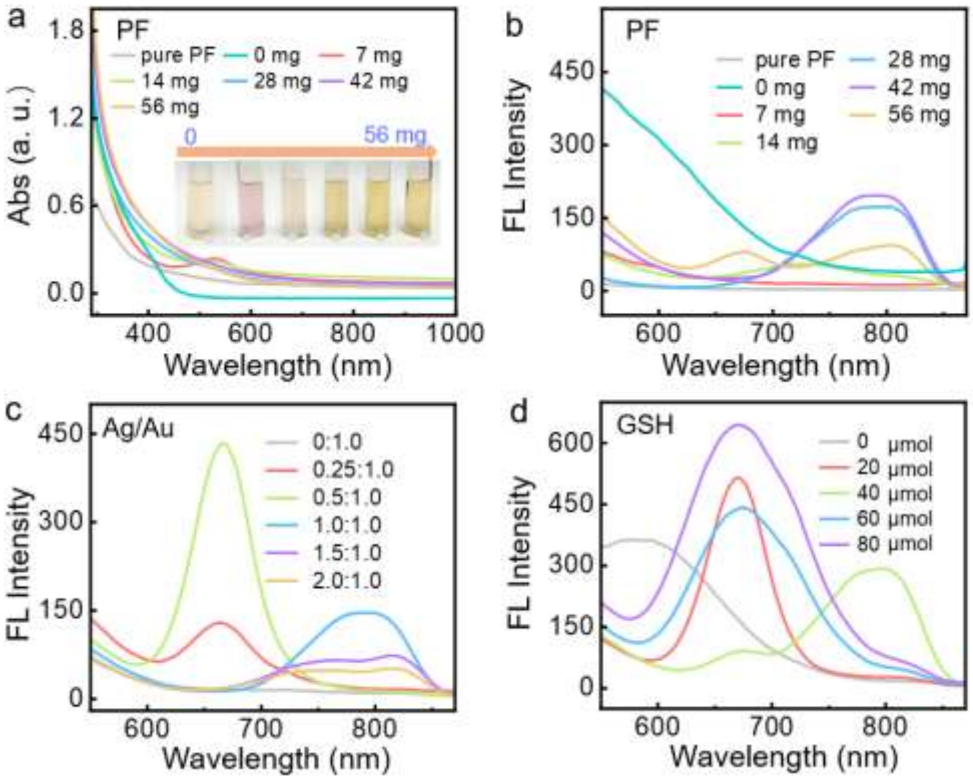

Figure S7. (a) Absorption spectra of AuNCs@PF (3.5 mg mL^{-1}) obtained by using different amounts of PF. Insets are the photographs of the AuNCs@PF (3.5 mg mL^{-1}) solution. Photoluminescence spectra of AuNCs@PF (3.5 mg mL^{-1}) under different reaction conditions: (b) changing the amounts of polymer PF while maintaining other synthetic parameters as the follows: the feeding ratio of Ag/Au was 1.0 and the dosage of GSH was 40 µmol; (c) varying the different feeding ratios of Ag/Au (Ag:Au) under fixed amount of Au element and variation of Ag salt. PF = 28 mg; GSH = 40 µmol; (d) different dosages of GSH with fixed mass of PF (28 mg) and feeding ratio of Ag/Au (Ag:Au = 1.0).

Firstly, the amount of PF, as the soft template during the growth of nanocluster, can impose significant effect on the photoluminescence of resulting nanoclusters. When the PF amount was less than 14 mg, gold nanoparticles with clear localized surface plasmon resonance (LSPR) absorption peaks at around 520 nm, as depicted in **Figure S7a**, could be observed. When using an appropriate amount of PF (28 mg), 810 nm-emitting gold nanoclusters could be obtained. In term of Ag/Au ratio, in the absence of Ag, there was no emission over 600 nm (**Figure S7c**). When the ratio was in the range from 0 to 0.5, an intensive and sharp emission peak around 676 nm emerged. However, when the ratio was higher than 1.0, a new broad emission peak appeared at around 810 nm. We anticipated that different Ag/Au ratios significantly affect the emissive moieties in the afforded Au NCs.

We then evaluated the effect of the amount of GSH, we assumed that when the amount of GSH was less than 40 µmol, GSH mainly acted as a reducing agent to promote the growth of the core of nanocluster, which resulted in a progressive red-shift of emission peak maxima. While, when the amount was over 40 µmol, as surface ligands, too much GSH would lead to an increase of surface coverage of GSH, which then induced a blue-shift of maximal emission.

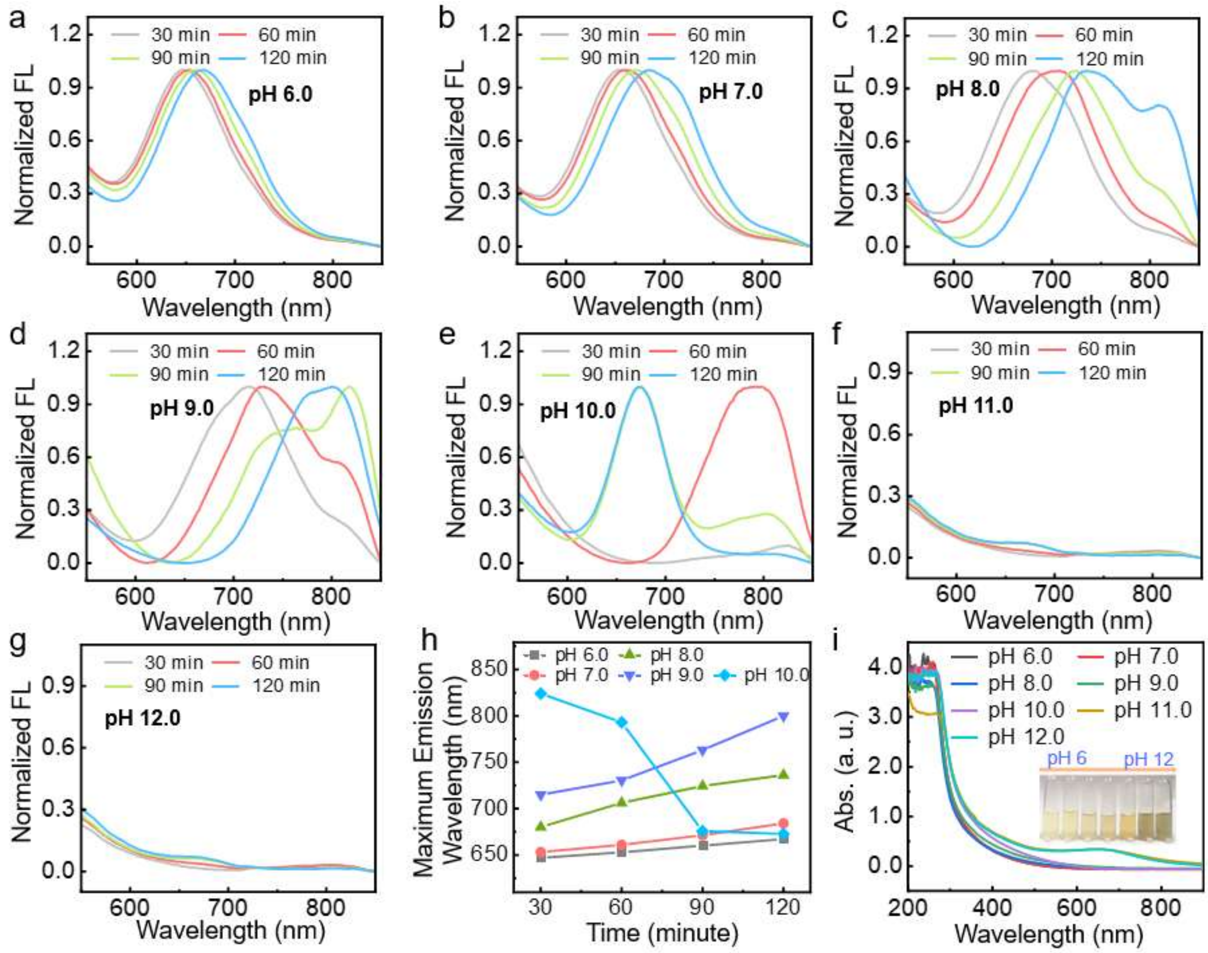

Figure S8. (a-g) The photoluminescence spectra of AuNCs@PF along with time under different pH conditions. (h) The variation of peak emission wavelength along with reaction time under different pH conditions. (i) The absorption spectra of AuNCs@PF synthesized under different pH conditions and heated at 80 °C for 2 h. It is clear that with the increase of pH value up to 11, the typical LSPR absorption of Au NPs was observed, implying the formation of Au NPs. Other synthetic conditions were as follows:the mass of PF was 28 mg, the feeding ratio of Ag/Au was 1.0 and the dosage of GSH was 40 μmol.

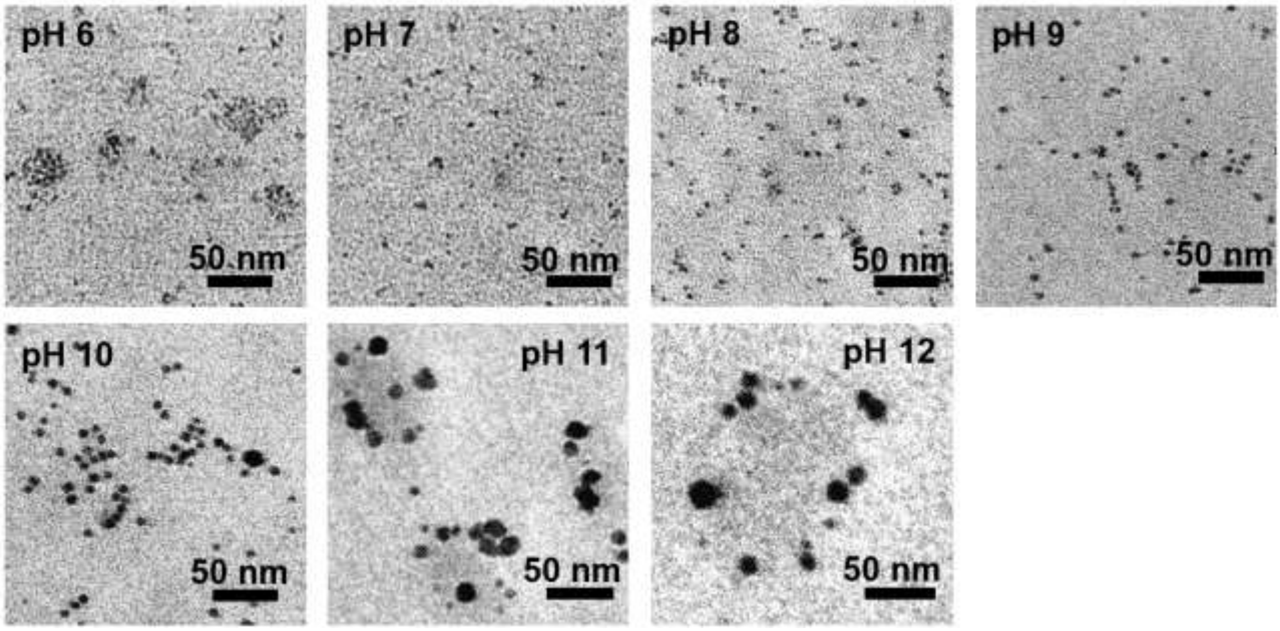

Figure S9. TEM images of AuNCs@PF prepared under different pH conditions. It is clear that the size of nanoclusters gradually increased with an increase of pH value and finally turned into nanoparticles, which is in accordance with the observation in the LSPR absorption shown in **Figure S8i**.

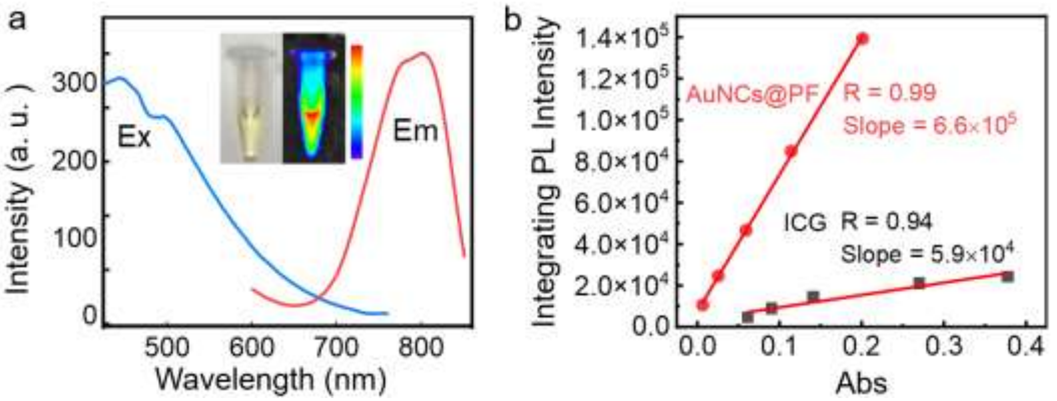

Figure S10. (a) Excitation and emission spectra of AuNCs@PF (5 mg mL^{-1}). The maximal excitation and emission wavelength were found to be 450 and 810 nm for AuNCs@PF, respectively. Inset photos were AuNCs@PF under daylight (left) and 450-nm light excitation (right). (b) The linear relationship of the integrated fluorescence intensity of AuNCs@PF and ICG. Near-infrared dye (ICG) at five different concentrations was used as reference to calculate the quantum yield of AuNCs. According to the previous report, the quantum yields of ICG in water is 2.5%[1]. The QYs for AuNCs@PF with peak emission at 810 nm was calculated to be 27.7%. Synthetic conditions of AuNCs@PF were as follows: 100 µL of $HAuCl_4$ solution (10 mM) was added into solution containing 28 mg of PF and stirred at room temperature for 10 min. The pH of the mixture was adjusted with NaOH to 9.0 to form Au-polymer complex and heated to 80 °C. After stirring for 2 h, 100 µL of $AgNO_3$ solution (10 mM) was added into the resulting solution and heated for another 1 h. Next, 40 µmol of GSH solution disolved in 2 mL water was added and heated for another 2 h to obtain AuNCs@PF.

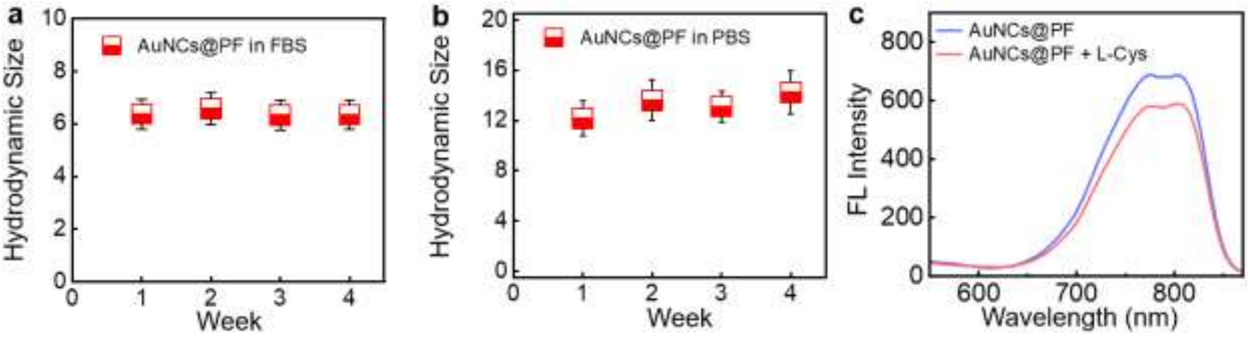

Figure S11. (a) The evolution of average DLS sizes of AuNCs@PF in fetal bovine serum (FBS) and (b) phosphate buffer saline (PBS) solution during 4 weeks. (c) The photoluminescent spectra of AuNCs@PF incubated with and without L-cysteine (1 mM) for 12 h. Unlike previously reported AuNCs, of which the luminescence intensities were readily quenched by biothiols such as L-cysteine, AuNCs@PF displayed excellent tolerance to the presence of high concentrations of biothiols.

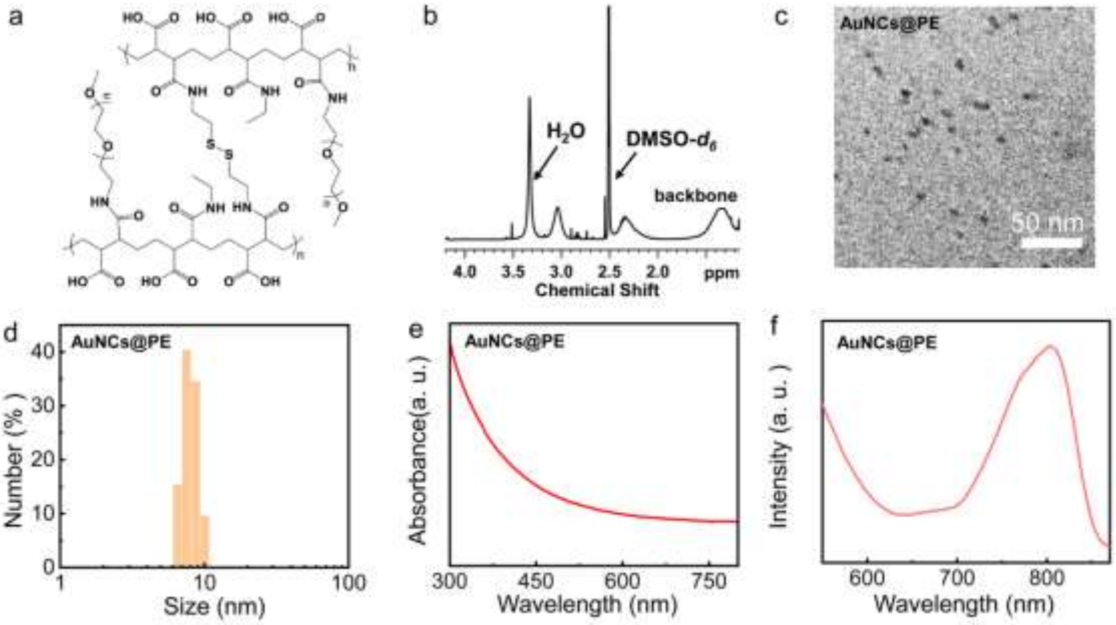

Figure S12. (a) Chemical structure and (b) 1H NMR spectrum of polymer PE, (c) TEM image, (d) DLS distribution, (e) absorption spectrum and (f) photoluminescent spectrum of AuNCs@PE. PE is the polymer functionalized with ethylamine instead of 2, 2, 2-trifluoroethylamine, therefore, no fluorinated side chains were introduced in the polymer matrix. In comparison with AuNCs@PF, the AuNCs@PE have the similar particle size, shape and luminescence properties.

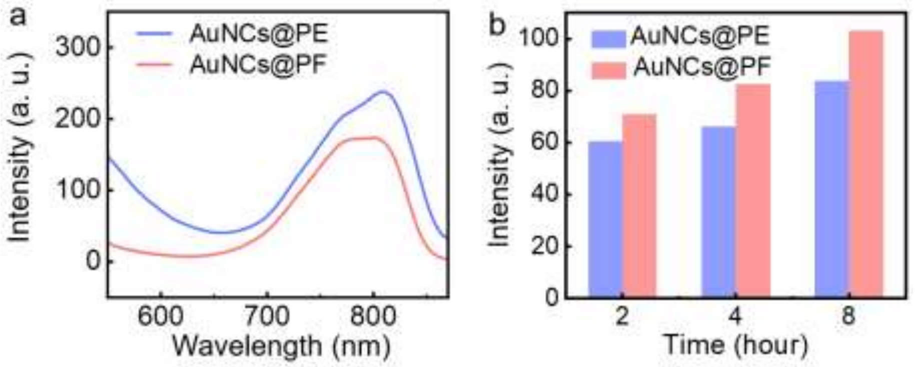

Figure S13. (a) The photoluminescent spectra of AuNCs@PE and AuNCs@PF, used in cell and tumor 3D tumor spheroids imaging experiments. The concentration of AuNCs@PE and AuNCs@PF colloidal solution is 3.5 mg/mL. (b) The luminescent intensity of 4T1 cells treated with AuNCs@PF and AuNCs@PE, respectively, at different time intervals.

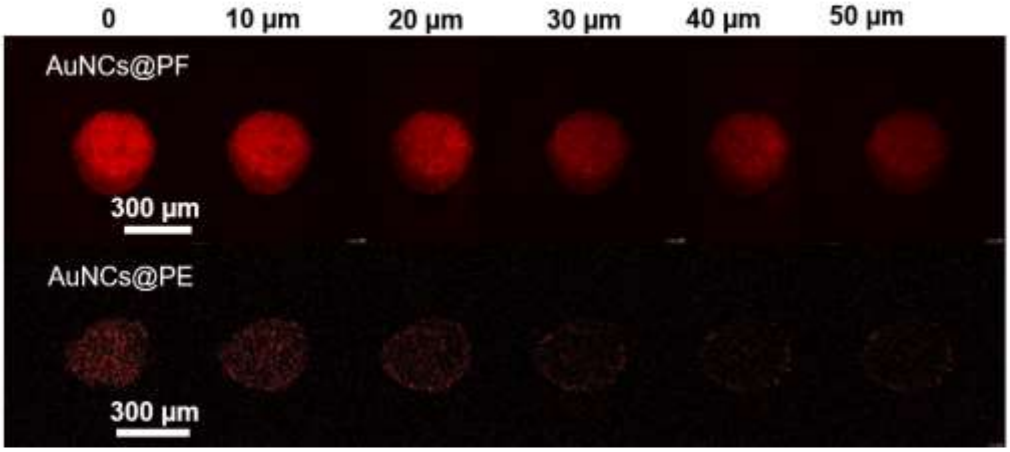

Figure S14. CLSM images of 3D tumor spheroids treated with AuNCs@PF and AuNCs@PE, respectively, at different imaging depths. All the results suggest that AuNCs@PF demonstrated better tumor penetrability than that of AuNCs@PE, due to the fluorinated polymer coating.

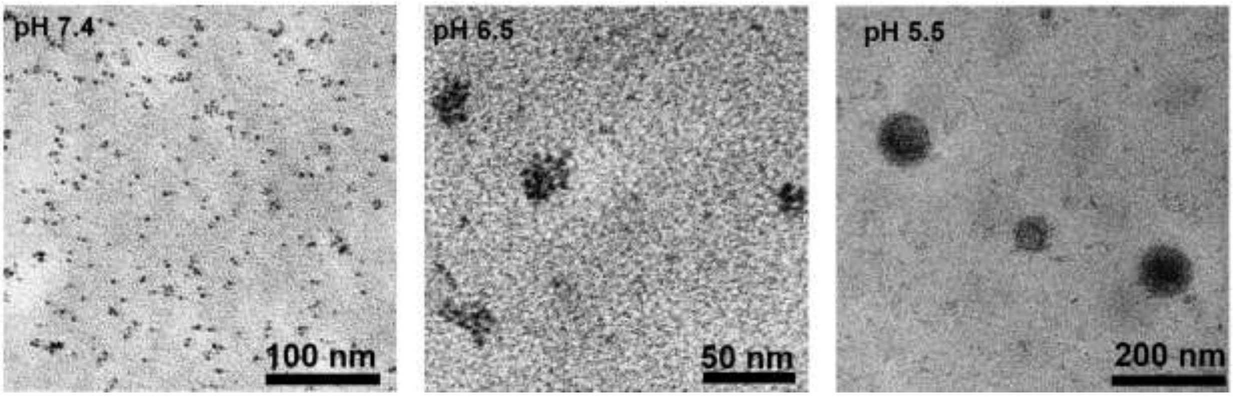

Figure S15. TEM images of AuNCs@PF under pH 7.4, 6.5 and 5.5 conditions, respectively. With the decrease of pH value from 7.4 to 5.5, the sizes of AuNCs@PF aggregates increased gradually to about 100 nm.

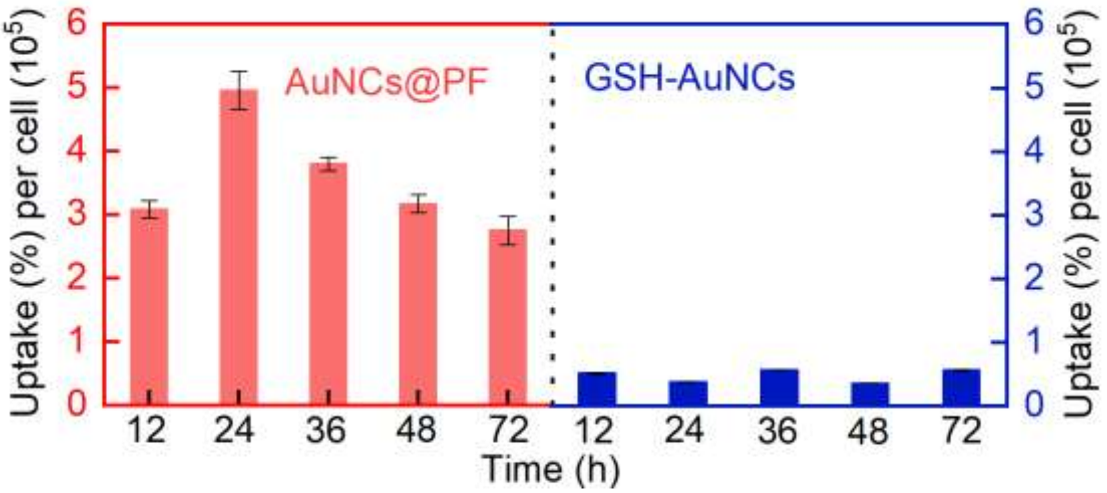

Figure S16. Cellular uptake of AuNCs@PF and GSH-AuNCs with different incubation time (12, 24, 36, 48 and 72 h). 200 μg/mL of AuNCs@PF and GSH-AuNCs were utilized for incubation with cells, where the Au content was 49.25 μg/mL and 88.65 μg/mL, respectively. The results suggested that though AuNCs@PF with lower Au content was employed, yet, higher Au content was retained in cells than that treated with GSH-AuNCs, demonstrating the high cell retention of AuNCs@PF.

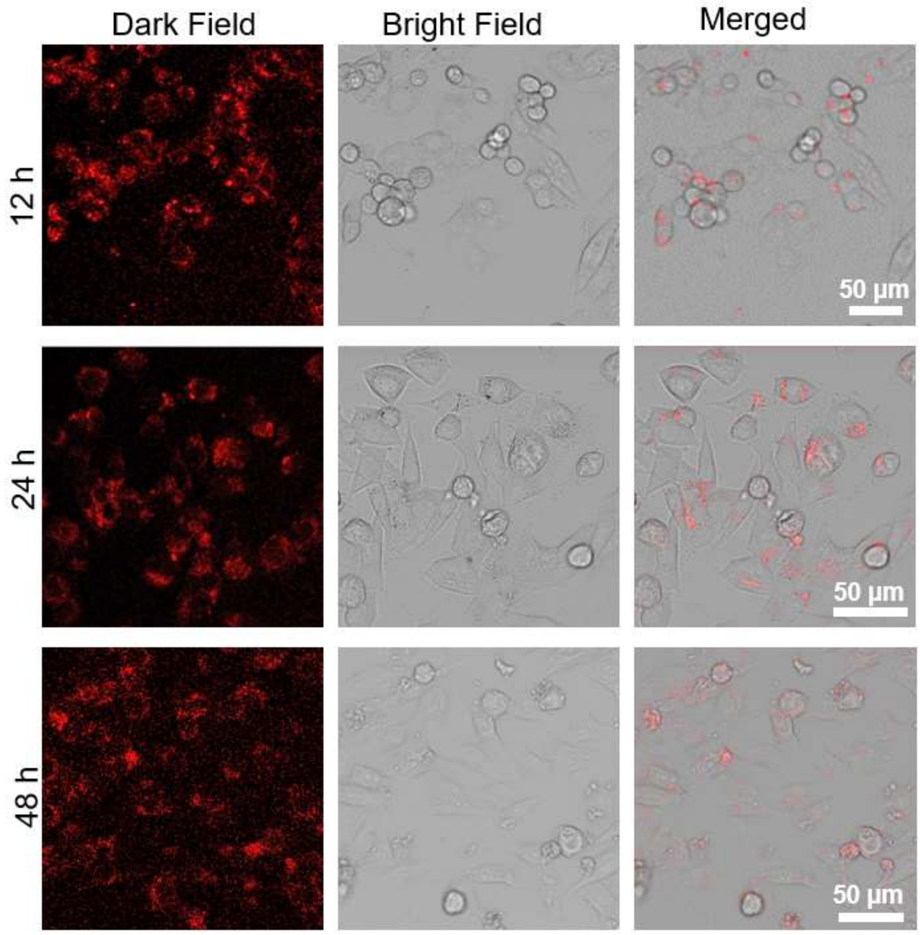

Figure S17. Cell imaging of AuNCs@PF with prolonged incubation time (12, 24 and 48 h). 200 μg/mL of AuNCs@PF was utilized for incubation with cells. This result suggested AuNCs@PF could be retained in cells for a long time due to acid induced aggregation.

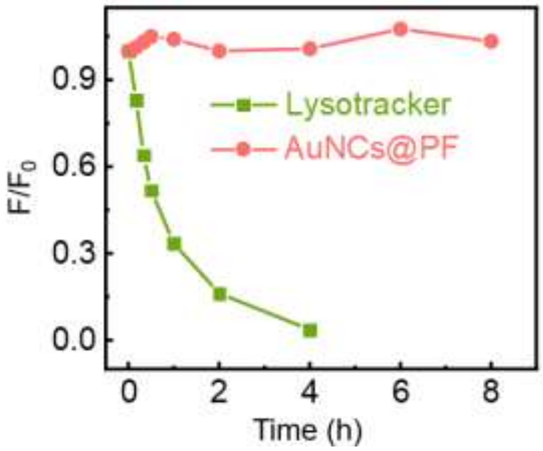

Figure S18. The variation in fluorescent intensity of lysosome probe LysoTracker Green DND-26 and AuNCs@PF under continuous 365-nm excitation.

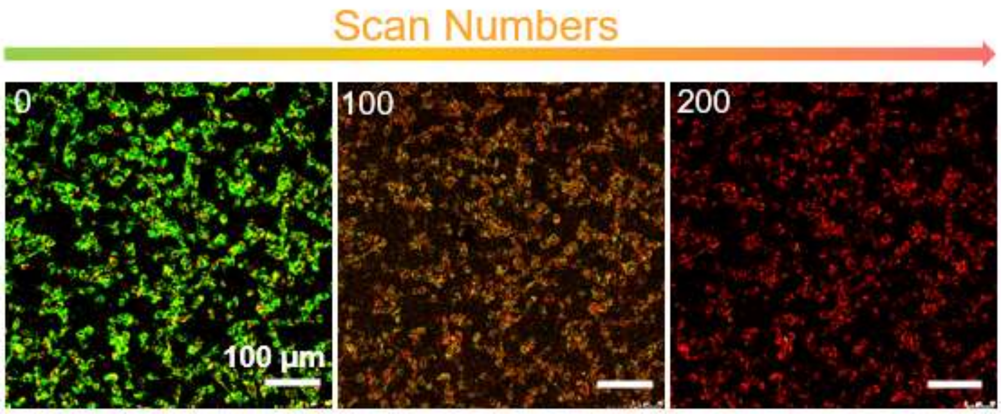

Figure S19. Overlaid confocal images of 4T1 cells treated with lysosome probe (LysoTracker Green DND-26) and AuNCs@PF under continuous scans with 488-nm laser irradiation. At first, the green fluorescence of lysosome probe is much brighter than the red fluorescence of AuNCs@PF, however, after scanning for 100 times, very weak green fluorescence was observed and the red fluorescence was well maintained. With 200 scans, the green fluorescence was almost totally quenched with only red fluorescence left. All the results suggested the high optical stability of AuNCs@PF.

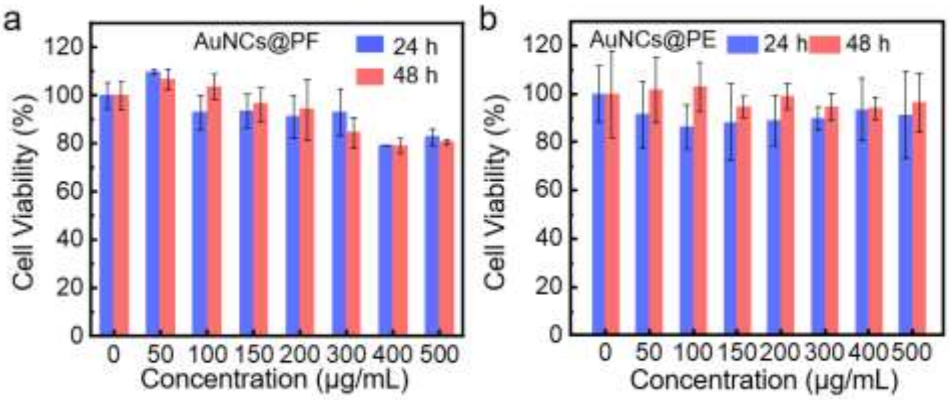

Figure S20. Cell viability of 4T1 cells incubated with AuNCs@PF (a) and AuNCs@PE (b) at various concentrations, respectively.

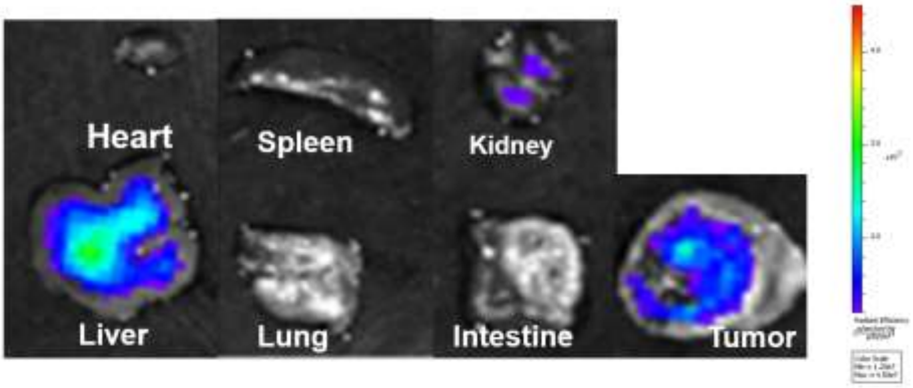

Figure S21. Fluorescent images of different organs and tumor obtained from mouse after intravenous injection with AuNCs@PF for 24 h. Note that the fluorescent intensity in tumor was different from that in Figure 4a due to different color scale.

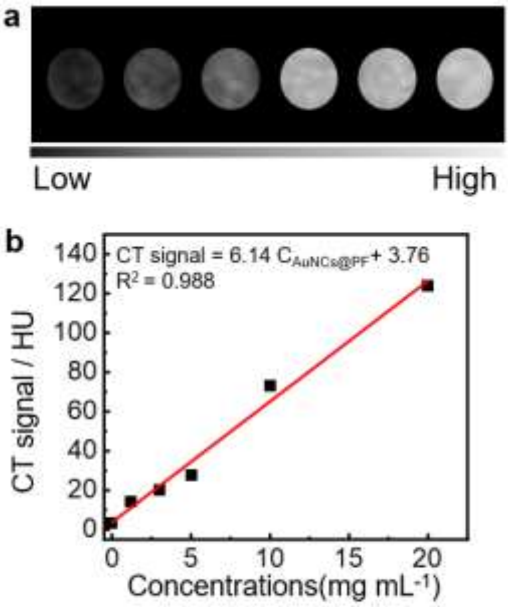

Figure S22. (a) *In vitro* CT images of AuNCs@PF solution with different concentrations. (b) The linear relationship between CT signal intensity *versus* sample concentrations.

Figure S22 showed the *in vitro* CT images of AuNCs@PF solution with different concentrations (0 – 20 mg mL^{-1}). The CT signal increased linearly with an increase of sample concentration, indicating the X-ray attenuation ability of AuNCs@PF was dependent on the amount of Au content.

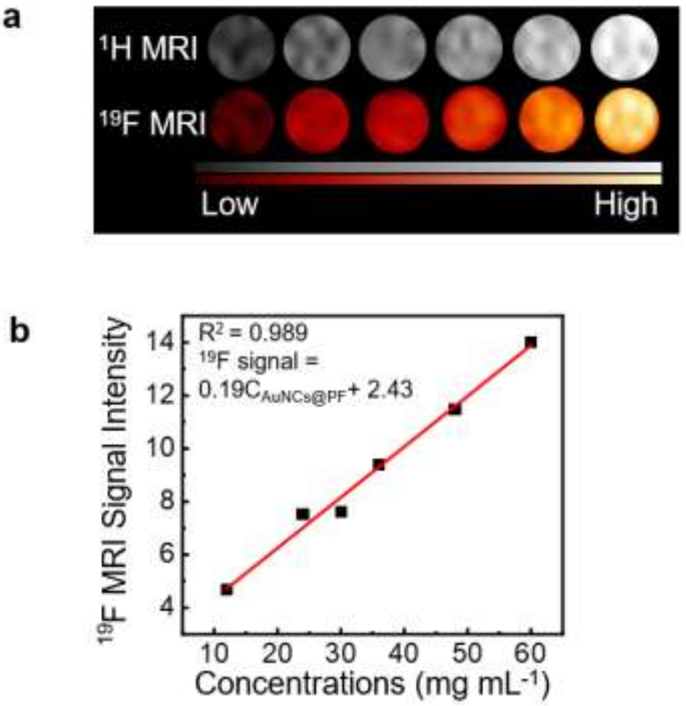

Figure S23. (a) *In vitro* ^{19}F/^{1}H MRI images of AuNCs@PF at different concentrations. (b) The linear relationship between ^{19}F MRI signal intensity *versus* sample concentration.

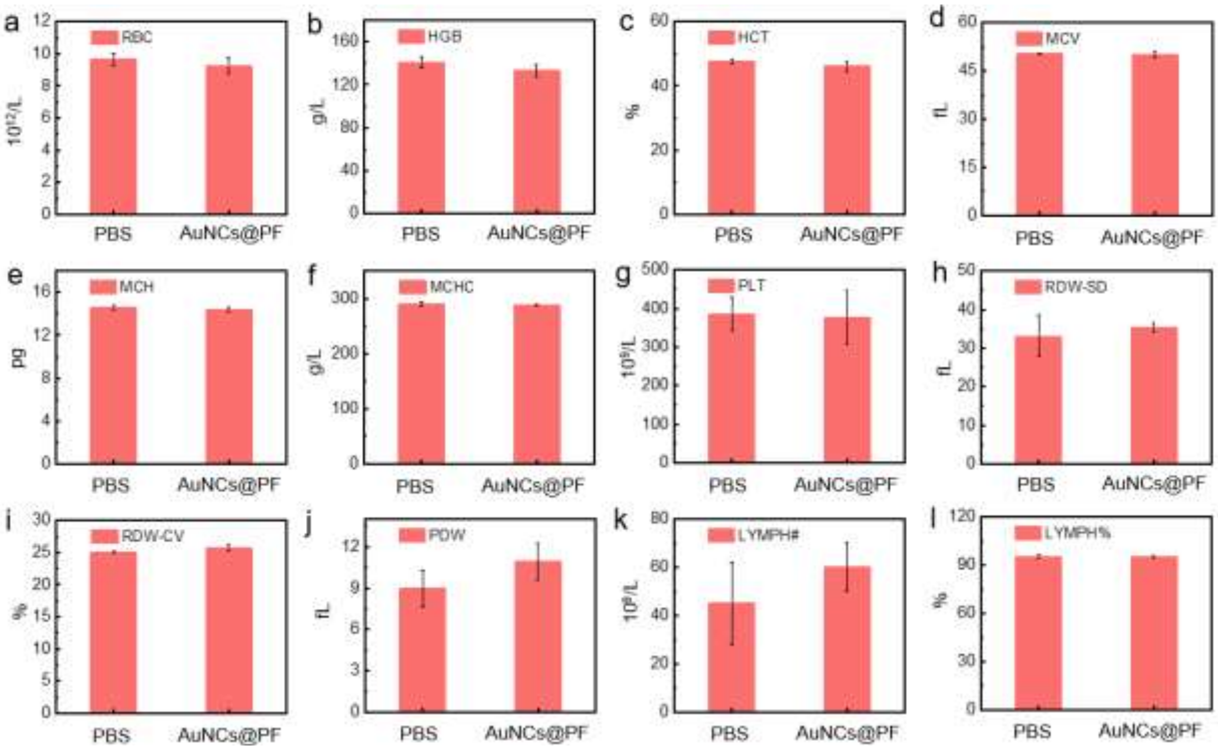

Figure S24. Blood biochemical and hematological analysis of the mice under the treatment of PBS and AuNCs@PF at 24 h post-injection. (a) RBC, red blood cells; (b) HGB, hemoglobin; (c) HCT, hematocrit; (d) MCV, mean capsular volume; (e) MCH, mean capsular hemoglobin; (f) MCHC, mean corpuscular hemoglobin concentration; (g) PLT, platelets; (h) RDW-SD, standard deviation of RBC distribution width; (i) RDW-CV, coefficient of variation of RBC distribution width; (j) PDW, platelet distribution width; (K) LYMPH#, absolute lymphocyte; (l) LYMPH%, lymphocyte percentage.

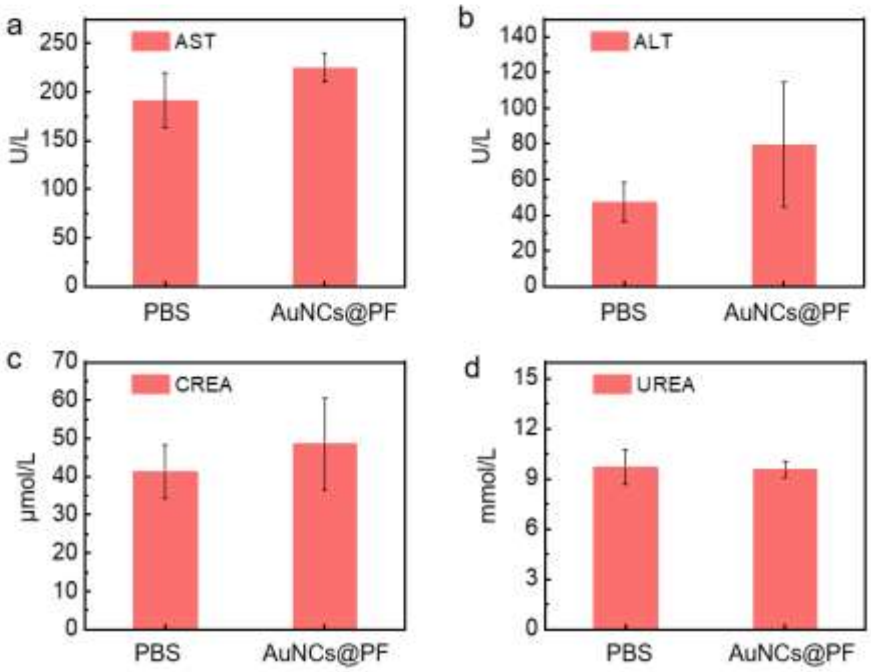

Figure S25. Blood biochemistry assays of liver and kidney function markers after intravenous injection of PBS and AuNCs@PF, respectively, for 24 h. Data are presented as the mean ± SD (*n* = 3). (a) AST, aspartate transferase; (b) ALT, alanine transferase; (c) CREA, creatinine; (d) UREA, urea.

Supporting Tables

Table S1. The variation in ^{19}F NMR signal intensity of modified PEMA prepared under different conditions.

**PEMA**	**2, 2, 2-trifluoroethylamine**	**Cystamine**	**mPEG-NH_2**	**^{19}F NMR signal intensity of PF (150 mg/mL)**
1.0 mmol	4.0 mmol	1.0 mmol	0	3295
1.0 mmol	4.0 mmol	1.0 mmol	0.2 mmol	9780
1.0 mmol	4.0 mmol	1.0 mmol	0.4 mmol	6870

*m*PEG-NH_2 here was used to alter the ^{19}F NMR signal intensity of PF. When PF was prepared without mPEG-NH_2, the ^{19}F NMR signal intensity was relatively low due to the hydrophobic aggregation of fluorine atoms, whereas, more mPEG-NH_2 used in preparing PF would compete with 2, 2, 2-trifluororethylamine during the ring-open reaction, which induced the decrease of ^{19}F NMR signal intensity. Thus, 0.2 mmol mPEG-NH_2 (10 mg) was chose to fabricate PF.

Table S2. Comparison of optical performance of AuNCs obtained in this work and literature

Sample	Excitation Wavelength (nm)	Emission wavelength (nm)	Quantum yields	Lifetime	References*
AuNCs-MOF	365	570	7.74%	-	Nanoscale [2]
AgNCs@ZIF-8	365	570	33.6%	9.18 μs	ACS. Appl. Mater. Interfaces [3]
AuNCs@GC	365	598	36.42%	-	Anal. Chem [4]
Au-GSH-PAH	450	600	25%	2.2 μs	ACS Nano [5]
MUA–AuNCs	302	607	13%	-	ACS. Appl. Mater. Interfaces [6]
$[Au_{25}(p\text{-}MBA)_{18}]^-$	365	620	6.2%	-	Agnew Chem Int Ed [7]
PCIE-AuNCs	312	645	4.3−4.8%	0.72−0.84 μs	Nano. Lett [8]
Au_{22}-Py	520	650	30%	1.45 μs	Nanoscale [9]
Au@AgNCs	520	667	15%	-	Nanoscale [10]
$Au_1Ag_{28}(LA)_{12}^{3-}$	485	675	1.7%	2.6 μs	ACS Nano [11]
K-AuNCs	480	680	12.4%	2.48 μs	ACS. Appl. Mater. Interfaces [12]
AuNCs	365	750	10.14%	300 ns	ACS Nano [13]
Ag-doped AuNCs	405	810	12%	0.99 μs	Chem. Mater[14]
AuNPs@CP	400	810	5.9%	-	Nano Res. [15]
PMIZ-AuNPs	-	810	1.79%	4.12 μs	ACS Nano [16]
Ag@AuNPs	-	810	1.03%	-	J. Phys. Chem. Lett[17]
$Au_{25}(SR)_{18}$	-	900	3.8%	-	Nano. Lett[18]
CD-AuNCs	808	1050	0.11%	0.17 μs	Agnew Chem Int Ed [19]
AuNCs	-	1050	1.9%	-	Agnew Chem Int Ed [20]
**AuNCs@PF**	**450**	**810**	**27.7%**	**4.5 μs**	**This work**

*detailed information of the related references are presented in the reference list.

References

[1] T. Jin, S. Tsuboi, A. Komatsuzaki, Y. Imamura, Y. Muranaka, T. Sakata, H. Yasuda, *Med.Chem.Comm.* **2016**, *7*, 623-631.

[2] F. Cao, E. Ju, C. Liu, W. Li, Y. Zhang, K. Dong, Z. Liu, J. Ren, X. Qu, *Nanoscale* **2017**, *9*, 4128-4134.

[3] Q. Gao, S. Xu, C. Guo, Y. Chen, L. Wang, *ACS. Appl. Mater. Interfaces.* **2018**, *10*, 16059-16065.

[4] X. Bai, S. Xu, L. Wang, *Anal. Chem.* **2018**, *90*, 3270-3275.

[5] A. Yahia-Ammar, D. Sierra, F. Merola, N. Hildebrandt, X. Le Guevel, *ACS Nano* **2016**, *10*, 2591-2599.

[6] H. C. Chang, Y. F. Chang, N. C. Fan, J. A. Ho, *ACS Appl. Mater. Interfaces.* **2014**, *6*, 18824-18831.

[7] M. Zhu, Q. Yao, Z. Liu, B. Zhang, Y. Lin, J. Liu, M. Long, J. Xie, *Angew. Chem. Int. Ed.* **2021**.

[8] V. G. Deepagan, M. N. Leiske, N. L. Fletcher, D. Rudd, T. Tieu, N. Kirkwood, K. J. Thurecht, K. Kempe, N. H. Voelcker, A. Cifuentes-Rius, *Nano. Lett.* **2021**, *21*, 476-484.

[9] K. Pyo, V. D. Thanthirige, S. Y. Yoon, G. Ramakrishna, D. Lee, *Nanoscale* **2016**, *8*, 20008-20016.

[10] X. Dou, X. Yuan, Y. Yu, Z. Luo, Q. Yao, D. T. Leong, J. Xie, *Nanoscale* **2014**, *6*, 157-161.

[11] M. van der Linden, A. J. van Bunningen, L. Amidani, M. Bransen, H. Elnaggar, P. Glatzel, A. Meijerink, F. M. F. de Groot, *ACS Nano* **2018**, *12*, 12751-12760.

[12] X. Wang, Y. Wang, H. He, X. Ma, Q. Chen, S. Zhang, B. Ge, S. Wang, W. M. Nau, F. Huang, *ACS. Appl. Mater. Interfaces.* **2017**, *9*, 17799-17806.

[13] F. Aldeek, M. A. H. Muhammed, G. Palui, N. Zhan, H. Mattoussi, *ACS Nano* **2013**, *7*, 2509–2521.

[14] E. Oh, J. B. Delehanty, L. D. Field, A. J. Mäkinen, R. Goswami, A. L. Huston, I. L. Medintz, *Chem. Mater.* **2016**, *28*, 8676-8688.

[15] K. He, J. Zhu, L. Gong, Y. Tan, H. Chen, H. Liang, B. Huang, J. Liu, *Nano Research* **2021**, *14*, 1087-1094.

[16] Y. Tan, K. He, B. Tang, H. Chen, Z. Zhao, C. Zhang, L. Lin, J. Liu, *ACS Nano* **2020**, *14*, 13975-13985.

[17] Y. Wang, L. Liu, L. Gong, Y. Chen, J. Liu, *J. Phys. Chem. Lett.* **2018**, *9*, 557-562.

[18] Y. Chen, D. M. Montana, H. Wei, J. M. Cordero, M. Schneider, X. Le Guevel, O. Chen, O. T. Bruns, M. G. Bawendi, *Nano. Lett* **2017**, *17*, 6330-6334.

[19] X. Song, W. Zhu, X. Ge, R. Li, S. Li, X. Chen, J. Song, J. Xie, X. Chen, H. Yang, *Angew. Chem. Int. Ed.* **2021**, *60*, 1306-1312.

[20] W. Wang, Y. Kong, J. Jiang, Q. Xie, Y. Huang, G. Li, D. Wu, H. Zheng, M. Gao, S. Xu, Y. Pan, W. Li, R. Ma, M. X. Wu, X. Li, H. Zuilhof, X. Cai, R. Li, *Angew. Chem. Int. Ed.* **2020**, *59*, 22431-22435.
